# Multi-copper oxidases SKU5 and SKS1 coordinate cell wall formation using apoplastic redox-based reactions in roots

**DOI:** 10.1093/plphys/kiad207

**Published:** 2023-04-03

**Authors:** Chaofan Chen, Yi Zhang, Jianfa Cai, Yuting Qiu, Lihong Li, Chengxu Gao, Yiqun Gao, Meiyu Ke, Shengwei Wu, Chuan Wei, Jiaomei Chen, Tongda Xu, Jiří Friml, Junqi Wang, Ruixi Li, Daiyin Chao, Baocai Zhang, Xu Chen, Zhen Gao

**Affiliations:** College of Life Science and Fujian Provincial Key Laboratory of Haixia Applied Plant Systems Biology, Fujian Agriculture and Forestry University, Fuzhou, Fujian 350002, China; FAFU-UCR Joint Center for Horticultural Biology and Metabolomics, Haixia Institute of Science and Technology, Fujian Agriculture and Forestry University, Fuzhou, Fujian 350002, China; College of Life Science and Fujian Provincial Key Laboratory of Haixia Applied Plant Systems Biology, Fujian Agriculture and Forestry University, Fuzhou, Fujian 350002, China; FAFU-UCR Joint Center for Horticultural Biology and Metabolomics, Haixia Institute of Science and Technology, Fujian Agriculture and Forestry University, Fuzhou, Fujian 350002, China; FAFU-UCR Joint Center for Horticultural Biology and Metabolomics, Haixia Institute of Science and Technology, Fujian Agriculture and Forestry University, Fuzhou, Fujian 350002, China; FAFU-UCR Joint Center for Horticultural Biology and Metabolomics, Haixia Institute of Science and Technology, Fujian Agriculture and Forestry University, Fuzhou, Fujian 350002, China; FAFU-UCR Joint Center for Horticultural Biology and Metabolomics, Haixia Institute of Science and Technology, Fujian Agriculture and Forestry University, Fuzhou, Fujian 350002, China; State Key Laboratory of Plant Genomics, Institute of Genetics and Developmental Biology, The Innovative Academy of Seed Design, Chinese Academy of Sciences, Beijing 100101, China; National Key Laboratory of Plant Molecular Genetics, CAS Center for Excellence in Molecular Plant Sciences, Shanghai Institute of Plant Physiology and Ecology, Chinese Academy of Sciences, Shanghai 200032, China; College of Life Science and Fujian Provincial Key Laboratory of Haixia Applied Plant Systems Biology, Fujian Agriculture and Forestry University, Fuzhou, Fujian 350002, China; FAFU-UCR Joint Center for Horticultural Biology and Metabolomics, Haixia Institute of Science and Technology, Fujian Agriculture and Forestry University, Fuzhou, Fujian 350002, China; College of Life Science and Fujian Provincial Key Laboratory of Haixia Applied Plant Systems Biology, Fujian Agriculture and Forestry University, Fuzhou, Fujian 350002, China; FAFU-UCR Joint Center for Horticultural Biology and Metabolomics, Haixia Institute of Science and Technology, Fujian Agriculture and Forestry University, Fuzhou, Fujian 350002, China; College of Life Science and Fujian Provincial Key Laboratory of Haixia Applied Plant Systems Biology, Fujian Agriculture and Forestry University, Fuzhou, Fujian 350002, China; FAFU-UCR Joint Center for Horticultural Biology and Metabolomics, Haixia Institute of Science and Technology, Fujian Agriculture and Forestry University, Fuzhou, Fujian 350002, China; FAFU-UCR Joint Center for Horticultural Biology and Metabolomics, Haixia Institute of Science and Technology, Fujian Agriculture and Forestry University, Fuzhou, Fujian 350002, China; Faculty of Cell Biology, Institute of Science and Technology Austria (IST Austria), Klosterneuburg 3400, Austria; Department of Biology, Institute of Plant and Food Science, Southern University of Science and Technology, Shenzhen, Guangdong 518055, China; Department of Biology, Institute of Plant and Food Science, Southern University of Science and Technology, Shenzhen, Guangdong 518055, China; National Key Laboratory of Plant Molecular Genetics, CAS Center for Excellence in Molecular Plant Sciences, Shanghai Institute of Plant Physiology and Ecology, Chinese Academy of Sciences, Shanghai 200032, China; State Key Laboratory of Plant Genomics, Institute of Genetics and Developmental Biology, The Innovative Academy of Seed Design, Chinese Academy of Sciences, Beijing 100101, China; FAFU-UCR Joint Center for Horticultural Biology and Metabolomics, Haixia Institute of Science and Technology, Fujian Agriculture and Forestry University, Fuzhou, Fujian 350002, China; FAFU-UCR Joint Center for Horticultural Biology and Metabolomics, Haixia Institute of Science and Technology, Fujian Agriculture and Forestry University, Fuzhou, Fujian 350002, China

## Abstract

The primary cell wall is a fundamental plant constituent that is flexible but sufficiently rigid to support the plant cell shape. Although many studies have demonstrated that reactive oxygen species (ROS) serve as important signaling messengers to modify the cell wall structure and affect cellular growth, the regulatory mechanism underlying the spatial-temporal regulation of ROS activity for cell wall maintenance remains largely unclear. Here, we demonstrate the role of the Arabidopsis (*Arabidopsis thaliana*) multicopper oxidase-like protein skewed 5 (SKU5) and its homolog SKU5-similar 1 (SKS1) in root cell wall formation through modulating ROS homeostasis. Loss of SKU5 and SKS1 function resulted in aberrant division planes, protruding cell walls, ectopic deposition of iron, and reduced nicotinamide adeninedinucleotide phosphate (NADPH) oxidase-dependent ROS overproduction in the root epidermis–cortex and cortex–endodermis junctions. A decrease in ROS level or inhibition of NADPH oxidase activity rescued the cell wall defects of *sku5 sks1* double mutants. SKU5 and SKS1 proteins were activated by iron treatment, and iron over-accumulated in the walls between the root epidermis and cortex cell layers of *sku5 sks1*. The glycosylphosphatidylinositol-anchored motif was crucial for membrane association and functionality of SKU5 and SKS1. Overall, our results identified SKU5 and SKS1 as regulators of ROS at the cell surface for regulation of cell wall structure and root cell growth.

## Teaser

Multi-copper oxidases SKU5 and SKS1 modulate reactive oxygen species homeostasis in root apoplast to regulate cell wall formation.

## Introduction

Plant cells are encased by a cell wall, which provides structural support for plant development and protects the plant against biotic and abiotic stresses. Primary cell walls are synthesized around the growing cell membrane with a strong but extensible polysaccharides matrix (Cosgrove and Jarvis 2012). The primary cell wall comprises 3 distinctive polysaccharides, including cellulose, hemicelluloses, and pectins, which constitute a multilayer nanostructure (Cosgrove 2016; Zhang et al. 2021b). Within the individual cell wall layers, stiff cellulose microfibrils form a reticulated, noncovalent network; hemicellulose binds noncovalently to cellulose and well-hydrated pectins, organizing into a gel-like matrix to support the stiff cellulose microfibrils network (Cosgrove 2016; Zhang et al. 2021b). During cell growth and plant development, plant cell walls undergo rapid but irreversible expansion, stimulating dynamic wall remodeling (O’Brien *et al*. 2012).

The generation of an appropriate amount of reactive oxygen species (ROS) promotes cell wall remodeling, which facilitates progression through the stages of plant development such as seed germination and fruit softening and contributes to defense responses (Fry *et al*. 2002; Schopfer and Liszkay 2006; Muller *et al*. 2009; Duan and Kasper 2011; Zhang *et al*. 2014; Jeevan Kumar *et al*. 2015). Apoplast-localized hydrogen peroxide (H_2_O_2_) serves as an oxidant to promote the oxidation of cell wall polysaccharides to cease cell growth (O’Brien *et al*. 2012; Karkonen and Kuchitsu 2015). Hydroxyl radical (OH·), another type of ROS, is a powerful agent to stimulate cell-wall loosening by nonenzymic scission within the cell wall polysaccharides chain (Fry 1998; Fry *et al*. 2001; Karkonen and Kuchitsu 2015). Plant respiratory burst oxidase homologs (RBOHs) are critical enzymes that function as ROS-producing reduced nicotinamide adeninedinucleotide phosphate (NADPH) oxidases on the plasma membrane (PM), fine-tuning various aspects of physiological processes required for plant growth, including pollen hydration and germination, stomatal closure, primary root development and elongation, lateral root emergence, and root hair tip growth (Foreman *et al*. 2003; Krieger *et al*. 2016; Orman-Ligeza *et al*. 2016; Watkins *et al*. 2017; Chapman *et al*. 2019; Liu *et al*. 2021). RBOHs have a core C-terminal region, which contains transmembrane domains and the functional oxidase domain responsible for superoxide (O_2_^·−^) generation (Suzuki *et al*. 2011). The O_2_^·−^ produced by RBOHs induces OH·-dependent cell-wall loosening, which is necessary for root cell elongation (Foreman *et al*. 2003; Dunand *et al*. 2007; Orman-Ligeza *et al*. 2016). During plant growth, the generation of a high amount of ROS is often stimulated by the over-accumulation of heavy metals, leading to the formation of an anisotropic cell pattern and root growth restriction (Muller *et al*. 2015; Wu *et al*. 2020). Although ROS molecules have been well-characterized to serve as pleiotropic physiological signaling agents (Sies and Jones 2020), the regulatory mechanism of apoplastic ROS for cell wall modification remains largely unknown.

Iron and its derivatives (e.g. heme or iron-sulfur [Fe-S] clusters) act as a component of co-factors of ROS-producing enzymes such as NADPH oxidase, cytochrome P450 enzymes, lipoxygenases, and subunits of the mitochondrial electron transport chain (Porta and Rocha-Sosa 2002; Jain and Connolly 2013; Dixon and Stockwell 2014; Xu *et al*. 2015; Wang *et al*. 2018). Iron also plays an important role in the active site of ROS-detoxifying enzymes such as catalase and ascorbate peroxidase (Anjum *et al*. 2016). Apart from the coordinated form, iron also actively participates in redox-based reactions via redox cycling between the Fe^2+^ and Fe^3 +^ forms (Kosman 2010). Under certain conditions, a high level of Fe^2+^ catalyzes the reaction between O_2_^·−^ and H_2_O_2_ to produce toxic ROS, including the strongly reactive OH· molecule (Halliwell and Gutteridge 1985; Chen and Schopfer 1999). Thus, excess iron accumulation in plants often results in ROS overproduction and consequent oxidative stress responses (Caro and Puntarulo 1996; Pekker *et al*. 2002; Ravet *et al*. 2009; Grillet *et al*. 2018). Recent studies have shown that excess iron and ROS in the root apoplast leads to the formation of an abnormal cell wall pattern and inhibition of root growth under a phosphate deficiency or ammonium supplementation background (Muller *et al*. 2015; Zheng *et al*. 2019; Liu *et al*. 2022). Thus, to avoid ROS over-accumulation in the plant cell wall, the spatial and temporal regulation of iron and ROS concentrations needs to be tightly controlled.

In this study, we identified the multicopper oxidase-like proteins skewed 5 (SKU5) and SKU5-similar 1 (SKS1) as regulators that maintain the ROS level at the cell surface and modulate cell wall formation. SKU5 and SKS1 are associated with PM via a glycosylphosphatidylinositol (GPI)-anchored motif, and the SKU5 and SKS1 protein levels could be stimulated by iron. Deficiency of SKU5 and SKS1 caused ectopic ROS and iron accumulation, particularly in the cell walls between the root epidermis–cortex and cortex–endodermis junctions, thereby resulting in aberrant division planes and cell wall protrusions. We further found that an respiratory burst oxidasehomologues C (RBOHC)-mediated ROS burst is required for appropriate SKU5/SKS1 regulation on cell walls. Our results provide a regulatory module involving SKU5 and SKS1 for cell wall formation, thereby extending our understanding of ROS homeostasis in the plant cell wall.

## Results

### SKU5 and SKS1 are necessary for normal root cell wall formation

**Figure 1.**
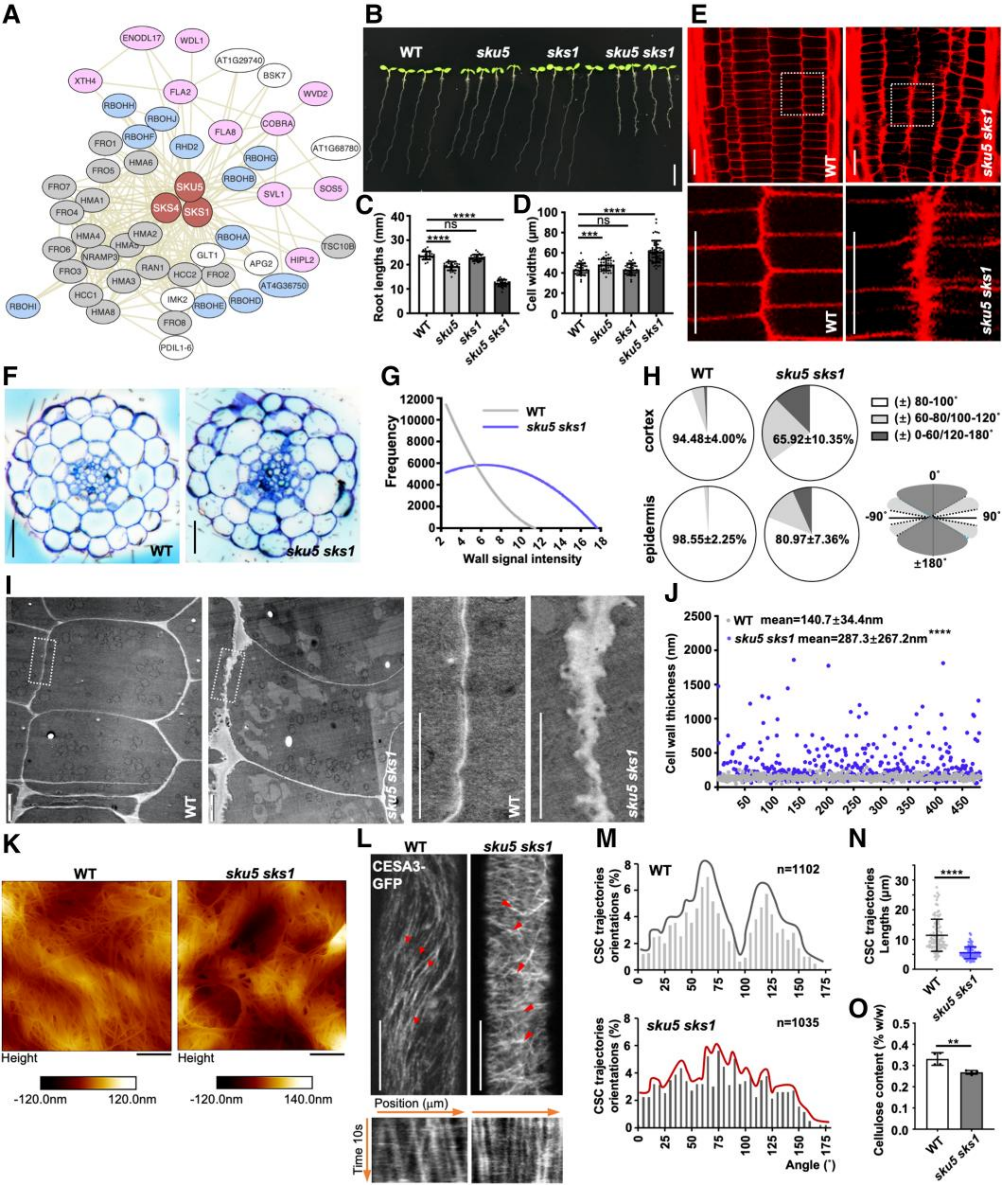
*sku5 sks1* exhibits disordered cell arrangement and defective cell wall structure in the primary root. **A)** Co-expressed network related to SKU5. **B to D)** Roots of 6-day-old WT, *sku5*, *sks1*, and *sku5 sks1* seedlings were observed and quantified as primary root length (C) and cell width (D) (C, from left to right, *n* = 25, 23, 26, and 28, **D)** from left to right, *n* = 37, 38, 37, and 51). **E, G)** Root meristematic zone of WT and *sku5 sks1* were stained by PI and the distribution of PI-indicated wall widths signal intensity was analyzed (G). The images in the bottom panels displayed 4× enlarged views of boxed areas in the original images (E). (G, *n* = 15 plants for each sample). **F)** Cross-sectioning was performed in root meristematic zones of WT and *sku5 sks1* seedlings. **H)** According to the deviated angles of the cell periphery perpendicular to the growth axis, root cells with different wall angles were grouped into 3 categories: (±) 80–100°, (±) 60–80°/100–120°, (±) 0–60°/120–180°. The percentage of different types of root cells was calculated in the cortex and epidermis layers of WT and *sku5 sks1* (WT, *n* = 33; *sku5 sks1*, *n* = 30). **I, J)** Cell wall morphology of WT and *sku5 sks1* root tips was visualized by TEM. The images in the right panels displayed 5× enlarged views of boxed areas in the original images (I). The Longitudinal cell wall thickness was measured in the root meristematic zone of WT and *sku5 sks1* based on TEM images (J) (*n* = 484 for each sample from 35 cells). **K)** AFM images show cellulose microfibrils on the cell wall of WT and *sku5 sks1* primary root cells. **L to N)** CESA3-labeled CSC trajectory was visualized in the hypocotyl of 3-day-old dark-grown WT and *sku5 sks1* (L). CSC trajectories orientations (M) and lengths (N) were measured. Arrows indicate the dynamics of CSC particles. The gray and red lines indicate distribution trends of CSC trajectories orientations in WT and *sku5 sks1*. The bottom panels showed the trajectory of CSC particles which were simulated by the maximum projection of image series (L) (M, *n* = 1,102 for WT, *n* = 1,035 for *sku5 sks1*; (N) *n* = 100 for each column). **O)** Cellulose level was measured in 7-day-old WT and *sku5 sks1* roots, respectively (*n* = 4 for each column). Scale bar, 5 mm (B), 20 µm (E), 25 µm (F), 2 µm (I), 200 nm (K), and 5 µm (L). Error bar = S.D. *P*-values were determined by two-tailed Student's t-test (C, D, J, N, O) (**P < 0. 01; ***P < 0. 001; ****P < 0.0001; ns, not significant).

A previous study showed that the Arabidopsis (*Arabidopsis thaliana*) *sku5* mutant displays a skewed root phenotype, which is likely caused by the mis-alignment of cellulose microfibrils (Sedbrook *et al*. 2002). Therefore, to further investigate whether SKU5 is involved in cell wall formation, we performed an unbiased analysis of the *SKU5* co-expression relationships to identify modules of co-expressed genes. The co-expression network showed that *SKU5* expression is closely associated with that of its homologs *SKS1* and *SKS4* (Fig. 1A). The ROS-producing genes *RBOHs*; metal uptake and translocation-related genes, including ferric-chelate reductases, heavy metal ATPases, and natural resistance-associated macrophage protein; and several well-known cell wall regulators, including *COBRA* and fasciclin-like arabinogalactan were co-expressed with *SKU5*, implying a potential relationship of SKU5/SKS with metal homeostasis and redox oxidation (Fig. 1A; Supplemental Table S1).

In Arabidopsis, the SKS family comprises 19 members (Sedbrook *et al*. 2002). Based on sequence similarity, the *SKU5/SKS* gene family belongs to a subcluster of multicopper oxidases (MCOs) and shows the highest sequence similarity with laccases (*LACs*), ascorbate oxidase (*AO*), low phosphate root, and others (Supplemental Fig. S1A). *SKS1* and *SKS2* exhibit the highest sequence similarity with *SKU5* (Supplemental Fig. S1A). Among them, *SKU5* and *SKS1* showed higher transcript levels than those of *SKS2* in the Arabidopsis root (Supplemental Fig. S1B), suggesting that SKU5 and SKS1 might primarily function in root development. However, *sku5* and *sks1* single mutants did not show any obvious cell wall-related defects, as detected by propidium iodide (PI) staining (Supplemental Fig. S1, C and E). We thus obtained *sku5 sks1* double mutant for further analyses.

Compared with wild type (WT), *sku5 sks1* double mutant displayed severe defects in root development, such as shorter and thicker primary roots and an increased root hair density (Fig. 1, B to D, Supplemental Fig. S1, D and F). Furthermore, *sku5 sks1* mutant showed a disordered cell growth pattern around the quiescent center (QC) (Supplemental Fig. S1G) and unexpected cell wall thickening in the longitudinal wall of dividing cells at the meristematic region (Fig. 1E). Cross-sectional slicing of the root meristematic zone displayed radial swelling of the root cortical and epidermal cells (Fig. 1F). To profile the abnormal morphology of *sku5 sks1* mutant, the PI-labeled wall thickness was analyzed to reconstruct the thickness map using the local thickness plug-in in Fiji (Supplemental Fig. S1H) (Dougherty and Kunzelmann 2007; Rothschild *et al*. 2017; Nabuqi *et al*. 2020). As a result, *sku5 sks1* roots showed higher variability in relative PI signal widths compared to those of the WT (Fig. 1G). Moreover, according to the deviated angles of the cell periphery, which is perpendicular to the growth axis, we grouped the root cells into 3 categories, (±) 80–100°, (±) 60–80°/100–120°, and (±) 0–60°/120–180°, corresponding to normal, mild-defect, and severe-defect cell division types, respectively (Fig. 1H). WT roots contained 95% normal cells in the cortex or 99% normal cells in the epidermis layers, which decreased to 66% or 81% in *sku5 sks1* double mutant, respectively (Fig. 1H). These data indicated that SKU5 and SKS1 are involved in root cell wall formation and in orienting root cell division.

Further visualization of cell wall morphology by transmission electronic microscopy (TEM) in conjunction with high-pressure freezing was used to provide a true snapshot at the moment of freezing: compared with the relative smooth morphology of longitudinal walls in WT, unexpected wall protrusions and enlarged cell–cell adhesion areas were found in the longitudinal walls of *sku5 sks1* mutant, particularly in the extracellular spaces between epidermis–cortex and cortex–endodermis layers (Fig. 1I). Quantification of the cell wall thickness of WT and *sku5 sks1* roots based on TEM images also showed thicker cell walls in the double mutant, which was consistent with the analysis of PI-labeled wall widths (Fig. 1J). These results are also in agreement with the previous characterization of *sku5 sks1 sks3* triple mutants that showed protruding cell walls (Zhou 2019).

The abnormal cell growth pattern in the *sku5 sks1* double mutant prompted us to explore whether the nanostructure of the cell wall was altered. Toward this end, we applied atomic force microscopy (AFM), a nanoscale imaging technique, to compare the cellulosic microfibrils on the cell walls of WT and *sku5 sks1* roots. The distribution of microfibrils was notably altered on *sku5 sks1* cell walls (Fig. 1K). Furthermore, uneven surfaces and pronounced cavities were present in *sku5 sks1* (Fig. 1K). Hence, the cellulosic microfibril network was clearly disorganized under SKU5 and SKS1 deficiency. The intensity of FM 4–64 dye at the PM is responsive to the quencher and is influenced by the nanoscale porosity of the cell wall; thus, the quenching efficiency can be used to indirectly reflect cell wall porosity (Liu *et al*. 2019). Therefore, we further evaluated the wall porosity of WT and *sku5 sks1* roots by quenching efficiency analysis. The *sku5 sks1* cell walls displayed higher quenching efficiency than that of WT, further supporting increased cell wall porosity in the *sku5 sks1* mutant (Supplemental Fig. S1, I and J).

The trajectory of the cellulose synthase complex (CSC) at PM is typically perpendicular to the cell expansion axis, reflecting the cellulosic microfibril orientation (Persson *et al*. 2007; Paredez *et al*. 2008). Next, we observed the movement of the cellulose synthase 3 (CESA3)–green fluorescent protein (GFP)-labeled CSC in WT and *sku5 sks1* double mutant using spinning-disc confocal microscopy (Fig. 1L). The orientations and lengths of the CSC trajectory were visualized and analyzed by the time-averaged projections of CSC particles' movement. *sku5 sks1* roots exhibited more random orientations and a truncated length of CSC trajectories compared with those of the WT (Fig. 1, M and N). Moreover, the mobility rate of CSC particles was reduced to 150 ± 6.2 nm/min in *sku5 sks1* mutant compared to 192 ± 3.4 nm/min in WT (Fig. 1L; Supplemental Fig. S2A). Given the disruption of the CSC trajectory in *sku5 sks1*, we further detected the cellulose content, which showed that the *sku5 sks1* mutant had a ∼20% lower cellulose level than that of WT (Fig. 1O).

It has been well-characterized that microtubules are co-aligned and parallel to the cellulose microfibrils, which determine the orientation of newly formed cellulose microfibrils (Li *et al*. 2015). We thus introduced the MT marker MAP4-GFP into the *sku5 sks1* mutant. In line with the above CSC trajectory orientation results, more random MT orientations were detected in the *sku5 sks1* mutant than in the WT (Supplemental Fig. S2B and C). Taken together, these findings demonstrated that SKU5 and SKS1 are required for the alignment of the cellulose microfibrils during cell wall formation.

### Elevated ROS levels cause the defective cell wall structure in *sku5 sks1* mutant

**Figure 2.**
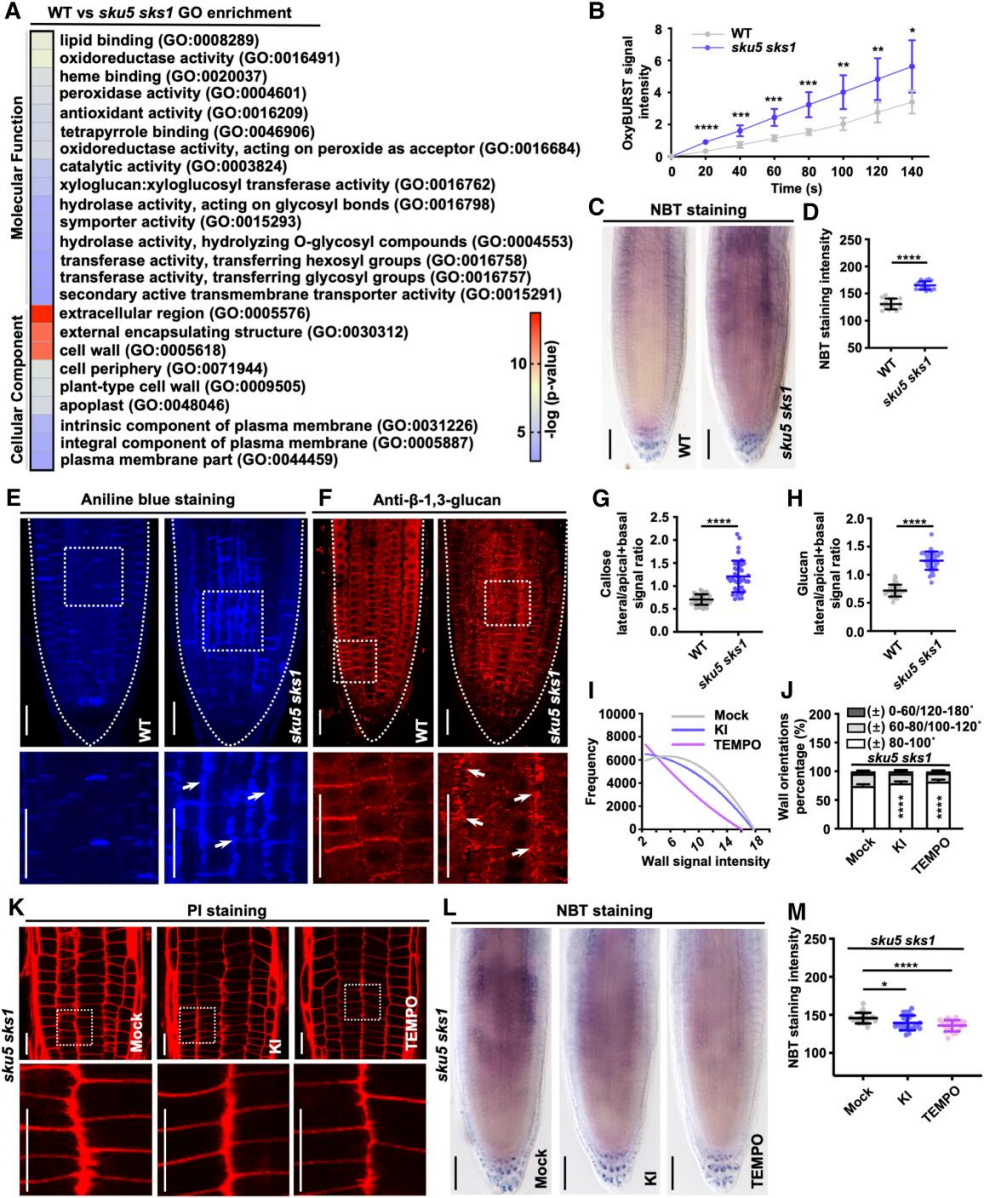
ROS level increased in the cell–cell junction area of *sku5 sks1* root meristem. **A)** GO items provide the transcriptome profiles of WT and *sku5 sks1* roots. **B)** Apoplastic ROS level was measured on the root surface of WT and *sku5 sks1* root tip by OxyBURST fluorescence (*n* = 6 for WT and *sku5 sks1*). **C, D)** O_2_^·−^ level was stained in WT and *sku5 sks1* roots by NBT staining (C) and the intensity was quantified in root meristematic zone (D) (D, *n* = 15 for each column). **E to H)** Callose deposition was observed in WT and *sku5 sks1* roots by aniline blue staining (E) and immunostaining against β−1,3-glucan antibody (F). The images in the bottom panels displayed the 3× enlarged views of boxed areas from the original images (E, F**).** Arrows highlight the deposited callose along the abnormal cell walls of *sku5 sks1*. The polarity of aniline blue-stained callose (G) and β-1,3-glucan-labelled callose (H) were quantified as the signal intensity ratio of lateral divided apical + basal signal (*n* = 40 for each column of (G, H**)**. **I to K)** Cell wall defects of *sku5 sks1* roots were restored after KI (750 µM) or 4-hydroxy-TEMPO (TEMPO) (2 mM) treatment (K). PI-stained wall widths signal intensity (I) and cell wall orientations (J) were quantified. The images in the bottom panels displayed 4× enlarged views of boxed areas in the original images (K). Asterisks indicate significant differences between the column of “± 80–100 °” (J) (I, *n* = 15 plants for sample; (J) *n* = 16 for each column). **L to M)** O_2_^·−^ level was decreased in *sku5 sks1* roots after KI (750 µM) or TEMPO (2 mM) treatment (L), and NBT staining intensity was quantified in root meristematic zone (M) (M, *n* = 22 for each column). Scale bar, 50 µm (C), 25 µm (E), 25 µm (F), 20 µm (K) and 50 µm (L). Error bar = S.D. P-values were determined by two-tailed Student's *t*-test (B, D, G, H, M**)** and two-way ANOVA (J) (*P < 0.05; **P < 0.01; ***P < 0.001; ****P < 0.0001).

To unravel the molecular mechanism by which SKU5 and SKS1 regulate cell wall formation, we conducted an RNA-sequencing experiment to compare the transcriptome profiles of the primary roots of WT and *sku5 sks1* mutant. Differentially expressed genes (DEGs) were identified based on a false discovery rate < 0.05 and |log_2_ fold-change| ≥ 1. According to the Gene Ontology (GO) enrichment of DEGs, oxidative regulation and extracellular component were among the top enriched GO terms, suggesting a potential association between SKU5/SKS1 and ROS burst during cell wall formation (Fig. 2A). A high ROS level has been shown to disrupt the stability of the polysaccharides matrix in the cell wall (Lindsay and Fry 2007), and previous studies have identified a role of SKS members in ROS regulation. For instance, the Arabidopsis *sks11 sks12* double mutant shows a decreased ROS level in the pollen tube (Duan *et al*. 2022), whereas the accumulation of ROS was reported in maize kernels in the *zmsks13* mutant (Zhang et al. 2021a). This background raised the question of whether the abnormal cell wall pattern observed in the *sku5 sks1* double mutant is caused by an elevated ROS level. To address this question, we employed an imaging approach based on the fluorogenic reagent OxyBURST Green H2HFF bovine serum albumin (BSA) to detect the ROS level in cell walls. OxyBURST is conjugated to BSA, which prevents the probe from penetrating the PM, enabling the monitoring of apoplastic ROS production by changes in OxyBURST fluorescence (Monshausen et al. 2007, 2009; Ivanchenko et al. 2013; Evans et al. 2016). We found that the fluorescence of OxyBURST rapidly increased along the root surface of the *sku5 sks1* mutant compared with that of WT (Fig. 2B, Supplemental Fig. S3A). We also detected global O_2_^·−^ accumulation in the root meristem zone using Nitroblue tetrazolium (NBT) staining (Jabs *et al*. 1996). *sku5 sks1* roots accumulated a higher level of O_2_^·−^ than those of WT (Fig. 2, C and D).

An apoplastic ROS burst stimulates local callose deposition at the cell wall (Benitez-Alfonso *et al*. 2011; Muller *et al*. 2015). Aniline blue staining and immunostaining with a β−1,3-glucan antibody are 2 approaches that are commonly used to visualize callose deposition. Accumulation of callose at the cell–cell junction zone of the epidermis–cortex and cortex–endodermis layers was detected in *sku5 sks1* mutant, whereas this unusual callose deposition was not found in WT (Fig. 2, E to H). Apoplastic ROS overproduction in *sku5 sks1* mutant roots, and callose deposition within cell–cell junctions along the longitudinal thickened cell wall in *sku5 sks1* mutant suggest an association between the protruding wall structure and ROS accumulation under a state of SKU5 and SKS1 deficiency.

To investigate if the cell wall defects in *sku5 sks1* roots were caused by ROS deposition, we applied ROS scavengers, including the H_2_O_2_ scavenger potassium iodide (KI) and the O_2_^·−^ scavenger 4-hydroxy-TEMPO (TEMPO), to counteract the excessive ROS from *sku5 sks1* roots. In comparison to the KI treatment, which slightly rescued the *sku5 sks1* phenotype, the application of TEMPO significantly restored the misaligned cell periphery and ROS over-accumulation in the *sku5 sks1* mutant (Fig. 2, I to M). Taken together, these results indicated that the accumulation of ROS molecules, especially O_2_^·−^, in the root apoplast is likely the major contributor to the cell wall deformation of the *sku5 sks1* mutant.

### The RBOH-mediated ROS burst acts downstream of SKU5/SKS1 to regulate cell wall formation

**Figure 3.**
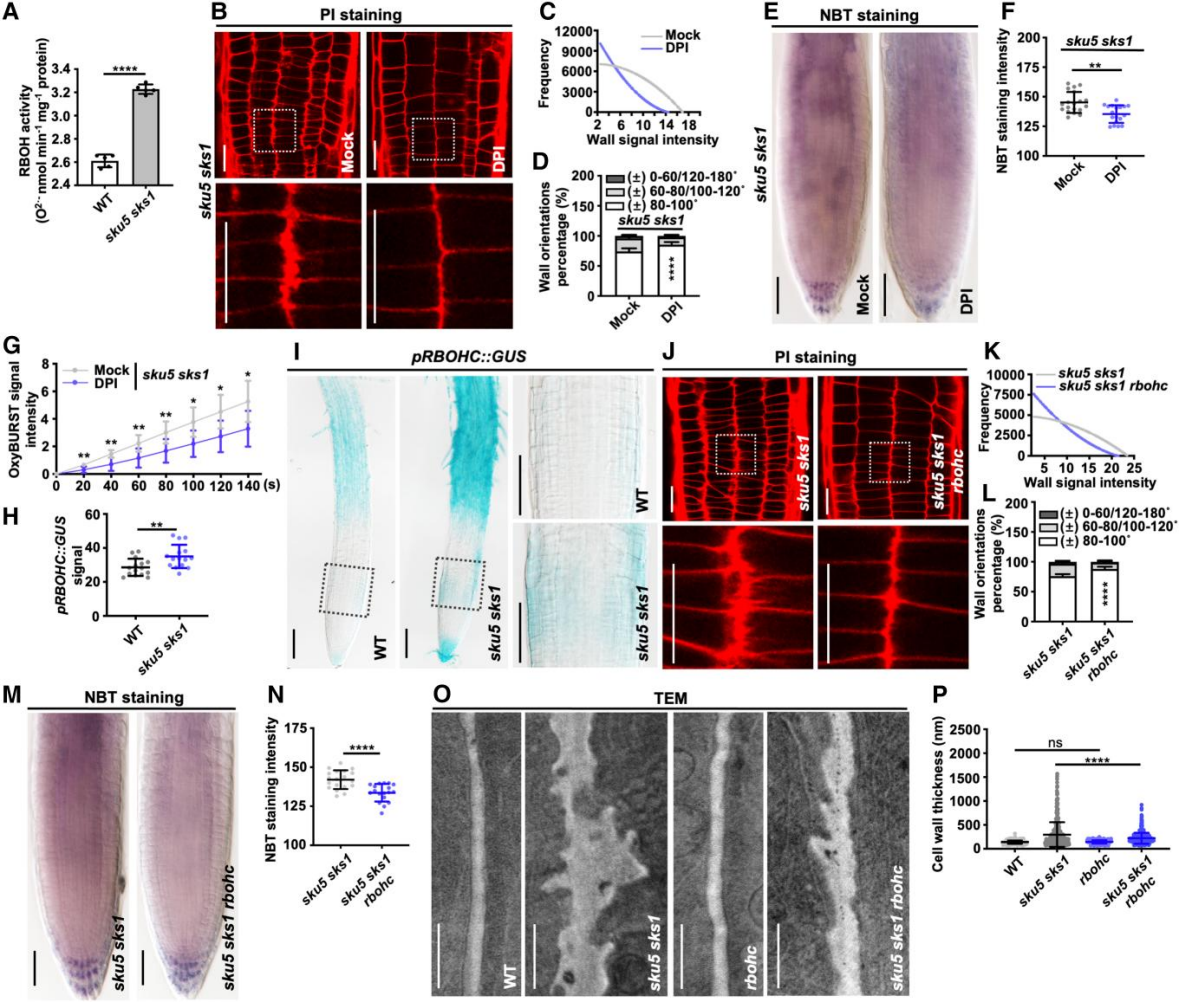
RBOH-dependent ROS overproduction causes the cell wall deformation of *sku5 sks1* roots. **A)** NADPH oxidase activity was measured in roots of WT and *sku5 sks1* mutant (*n* = 5 for each column). **B to D)** Defective cell walls of *sku5 sks1* roots were restored after DPI chloride treatment. PI-stained wall widths signal intensity and wall orientations were examined and quantified in mock and DPI (250 nM) treatment of *sku5 sks1* roots (B to D). The images in the bottom panels displayed 4 × enlarged views of boxed areas in the original images (B). Asterisks indicate significant differences between the column of “± 80–100 ˚” (D) (C, *n* = 15 plants for sample; (D**)***n* = 15 for each column). **E to F)** O_2_^·−^ level of *sku5 sks1* roots was stained by NBT with or without DPI (250 nM) treatment (E). NBT staining intensity was quantified in root meristematic zone (F) (F, *n* = 19 for mock, *n* = 18 for DPI). **G)** Apoplastic ROS was indicated by OxyBURST fluorescence on the surface of the meristem zone in mock and DPI (250 nM)-treated *sku5 sks1* (*n* = 7 for mock, *n* = 8 for DPI). **H, I)** The expression pattern of RBOHC was indicated by *pRBOHC::GUS* in the primary roots of WT and *sku5 sks1* mutants (I), and the GUS signal was quantified (H). The images in the right panels displayed 3× enlarged views of boxed areas in the original images (I) (H, *n* = 15 for each column). **J to L)** Root cell walls were visualized in *sku5 sks1* and *sku5 sks1 rbohc* by PI staining (J). PI-indicated wall widths signal intensity (K) and wall orientations (L) were quantified. The images in the bottom panels displayed 4× enlarged views of boxed areas in the original images (J). Asterisks indicate significant differences between the column of “± 80–100 ˚” (L) (K, *n* = 15 plants for each sample; L, *n* = 15 for each column). **M, N)** O_2_^·−^ level was visualized by NBT staining in *sku5 sks1* and *sku5 sks1 rbohc* mutant (M), and NBT staining intensity was quantified in root meristematic zone (N) (N, from left to right, *n* = 19 and 18). **O, P)** Cell wall morphology was visualized in root meristematic zone of WT, *sku5 sks1*, *rbohc*, and *sku5 sks1 rbohc* by TEM (O), and cell wall thickness was quantified (P) (P, *n* = 484 for each column from 35 cells). Scale bar, 20 µm (B), 50 µm (E), 100 µm (I), 20 µm (J), 50 µm (M) and 1 µm (O). Error bar = S.D. *P*-values were determined by two-tailed Student's *t*-test (A, F, G, H, N, P**)** and two-way ANOVA (D, L**)** (*P < 0.05; **P < 0. 01; ****P < 0.0001; ns, not significant).

RBOHs are predominant ROS donors on PM, which reduce O_2_ to O_2_^·−^ in the apoplast (Suzuki *et al*. 2011; Marino *et al*. 2012). To evaluate whether the apoplastic O_2_^·−^ deposition in *sku5 sks1* mutant is caused by elevated RBOH activity, we tested the NADPH oxidase activity in the primary roots of WT and *sku5 sks1* mutant. The NADPH oxidase activity was significantly increased in *sku5 sks1* compared with WT (Fig. 3A). We then applied RBOH inhibitor diphenyleneiodonium (DPI) to counteract the high NADPH oxidase activity in *sku5 sks1* roots. DPI treatment restored the thickening cell wall pattern and the misaligned cell periphery of *sku5 sks1* roots (Fig. 3, B to D). Excess O_2_^·−^ and apoplastic ROS in *sku5 sks1* roots were also eliminated by DPI (Fig. 3, E to G, Supplemental Fig. S3B). This implied that cell wall defects of *sku5 sks1* mutant are likely caused by RBOH-dependent ROS production.

To identify which RBOH member is activated in *sku5 sks1* mutant, RT-qPCR analysis of *RBOHs* was conducted in the root tip of WT and *sku5 sks1* (Supplemental Fig. S4B). The transcript levels of root-abundant RBOHs B–F were all elevated in the *sku5 sks1* mutant (Supplemental Fig. S4A and B). The *RBOH* gene expression map of the Arabidopsis root showed that *RBOHC* was particularly enriched in the root meristematic zone (Supplemental Fig. S4A). We thus introduced *pRBOHC::GUS* construct in WT and *sku5 sks1* mutant background to evaluate the expression of *RBOHC*. Compared with that of WT, *RBOHC* expression level was elevated in the roots of the *sku5 sks1* mutant (Fig. 3, H and I). In particular, highly activated expression of *RBOHC* was found in the root meristematic zone of the *sku5 sks1* mutant, which is in line with the region of defective walls (Fig. 3I).

We then generated *sku5 sks1 rbohc* triple mutants, in which loss of RBOHC function decreased the ROS level in the *sku5 sks1* background. Homozygous *sku5 sks1 rbohc* triple mutants significantly restored the defective phenotypes of *sku5 sks1*, including shorter roots, elevated ROS level, and wall thickening (Fig. 3, J to N; Supplemental Fig. S4, C to F). Measurement of cell wall thickness based on TEM images also confirmed that the cell wall protrusion defects in *sku5 sks1* mutant were restored in *sku5 sks1 rbohc* triple mutants (Fig. 3O to P). Therefore, R, B to H-dependent ROS overproduction is one of the key factors contributing to SKU5/SKS1-mediated cell wall formation. However, the loss-of-function of *RBOHC* only partially rescued the wall defects of *sku5 sks1*, suggesting the involvement of additional factors.

### SKU5 and SKS1 are iron-responsive proteins

**Figure 4.**
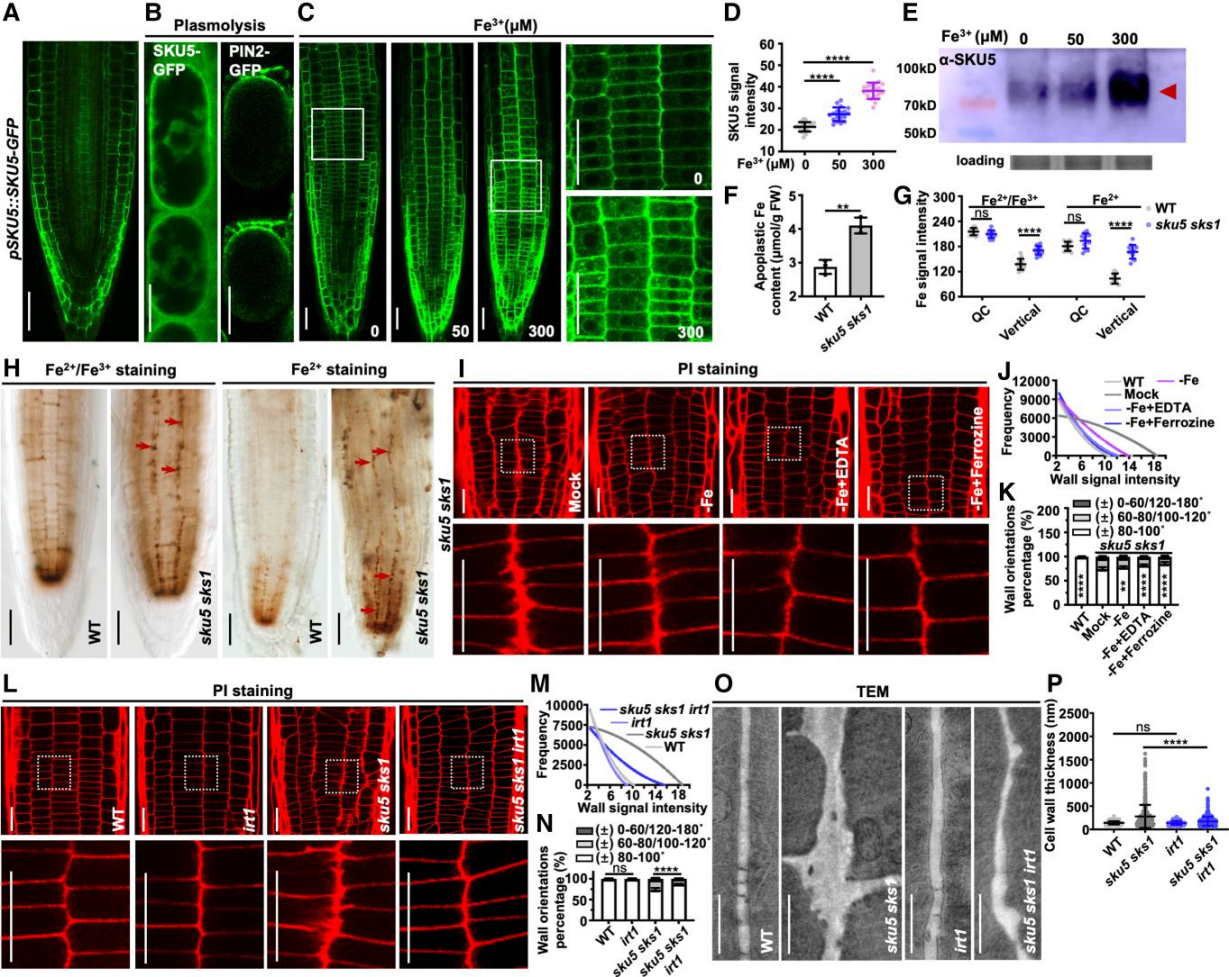
Elevated iron in root apoplast might cause cell wall deformation in *sku5 sks1*. **A)** Subcellular distribution of SKU5 protein. GFP signal was shown in *pSKU5::SKU5-GFP* root. **B)** Apoplastic SKU5 signals were detected by plasmolysis of *pSKU5::SKU5-GFP*. PM-localized *pPIN2::PIN2-GFP* was used as a control. **C, D)***pSKU5::SKU5-GFP* seedlings were grown on iron-deficient medium supplied with 0, 50, 300 μM Fe^3+^-EDTA (C), and SKU5 signal in root meristem was quantified (D). The images in the right panels displayed 3× enlarged views of boxed areas in the original images in the left panels (D, *n* = 19, 18, and 20). **E)** SKU5 protein level (labeled as an arrow) was detected in WT seedlings by western blotting (with SKU5 antibody) in iron-deficient medium supplied with 0, 50, 300 μM Fe^3+^-EDTA. **F)** Apoplastic Fe content was measured in WT and *sku5 sks1* roots (*n* = 3 for each column). **G to H)** Iron distribution was stained by Perls/DAB (left panels) or Turnbull/DAB (right panels) in WT and *sku5 sks1* (H). Arrows highlight the deposited iron along the defective cell walls of *sku5 sks1*. Signal intensity in the QC and longitudinal distribution were qualified (G). (G, *n* = 14, 14, 14, 14, 12, 12, 12, and 12). **I to K)** The defective cell walls of *sku5 sks1* were significantly restored in the iron-depleted medium. PI staining shows root cell morphology of *sku5 sks1* (I). *sku5 sks1* mutant was grown in iron-sufficient, iron-deficient, or iron-deficient medium supplied with 300 μM EDTA or 300 μM Ferrozine (I). PI-stained wall widths signal intensity (J) and wall orientations (K) were quantified. The images in the bottom panels displayed 4× enlarged views of boxed areas in the original images (I). Asterisks indicate significant differences between the column of “± 80–100 ˚” (K) (J, *n* = 15 plants for each sample, K, *n* = 16, 16, 15, 16, and 16). **L to N)** Cell morphology of WT, *irt1*, *sku5 sks1*, and *sku5 sks1 irt1* roots were observed by PI staining (L). PI-indicated wall widths signal intensity (M) and wall orientations (N) were quantified. Asterisks indicate significant differences between the column of “± 80–100 ˚” (N) (M, *n* = 15 plants for each sample, N, *n* = 16 for each column). **O, P)** Cell wall morphology was visualized in the root meristematic zone of WT, *sku5 sks1*, *irt1*, and *sku5 sks1 irt1* by TEM (O), and cell wall thickness was quantified (P) (P, *n* = 484 for each column from 35 cells). Scale bar, 50 µm (A), 20 µm (B), 20 µm (C), 50 µm (H), 20 µm (I), 20 µm (L), and 1 µm (O). Error bar = S.D. *P*-values were determined by two-tailed Student's t-test (D, F, G, P**)** and two-way ANOVA (K, N**)** (**P < 0. 01, ****P < 0.0001, ns, not significant).

SKU5 and SKSs belong to the family of GPI-anchor proteins that attach to the outer leaflet of the PM via the C-terminal GPI motif (Yeats *et al*. 2018). GPI-anchored proteins are localized at the PM–cell wall nexus, playing a crucial role in maintaining cell wall integrity (Yeats *et al*. 2018). The cell wall deformation and ROS overproduction of *sku5 sks1* roots prompted us to examine the subcellular localization of SKU5 and SKS1 proteins. We then obtained *pSKU5::SKU5-GFP* and *pSKS1::SKS1-GFP* transgenic plants. In the primary roots, SKU5-GFP signal was predominantly detected in the epidermis and cortex layers (Fig. 4A). SKS1-GFP was mainly detectable in the columella stem cells of the root tip, which was complementary to the SKU5 expression pattern (Supplemental Fig. S5A). Interestingly, the SKU5-GFP signal was notably detected in the apoplast by plasmolysis of *pSKU5::SKU5-GFP*, which was distinguished with transmembrane PIN-FORMED2 protein (Fig. 4B) (Ke *et al*. 2021). The unique localization pattern of the SKU5 protein implied that SKU5 might function in the apoplast for ROS production.

Apoplastic-deposited Fe^3+^ acts as a potential source of ROS production (Meguro *et al*. 2007; Kosman 2010). The distribution of SKU5 protein along the epidermis and cortex layers is consistent with the iron transport pathway, which occurs in the root laterally across the cortex and endodermis to the xylem (Kim and Guerinot 2007). Based on the co-expression network, the potential connection among iron, ROS, cell wall, and SKSs prompted us to examine the association between SKU5/SKS1 and iron (Fig. 1A). Therefore, *pSKU5::SKU5-GFP* seedlings were grown on medium supplemented with iron. SKU5-GFP signal was gradually elevated by the increase of exogenous iron, particularly along the PM (Fig. 4, C and D). We further detected the SKU5 protein level after iron treatment at different concentrations by western blotting. Exogenous iron substantially enhanced the SKU5 protein level (Fig. 4E). Similarly, treatment of exogenous iron on the *pSKS1::SKS1-GFP* roots resulted in an increased level of SKS1 protein (Supplemental Fig. S5B and C). Therefore, both SKU5 and SKS1 are iron-responsive proteins.

To investigate whether the cell wall deformation in *sku5 sks1* double mutant is related to the apoplastic iron accumulation, we measured the apoplastic iron content in *sku5 sks1* roots. The content of apoplastic iron in *sku5 sks1* roots was increased by more than 40% compared with that of WT (Fig. 4F). Moreover, Turnbull/diaminobenzidine (DAB) staining was applied to label Fe^2+^, and Perls/DAB staining was used to label the major Fe^3+^ and minor Fe^2+^ forms (Meguro *et al*. 2007; Roschzttardtz *et al*. 2009; Gutierrez-Alanis *et al*. 2017). Both Fe^3+^ and Fe^2+^ accumulated in the QC region of *sku5 sks1* and WT roots (Fig. 4, G and H). In comparison to that of WT, *sku5 sks1* roots displayed additional deposition of Fe^3+^ and Fe^2+^ along the longitudinal cell walls, which was consistent with the thickening cell wall in the double mutant (Fig. 4, G and H). To verify whether the defective cell wall in *sku5 sks1* mutant is caused by ectopic iron accumulation, WT, and *sku5 sks1* plants were grown on an iron-sufficient medium, iron-deficient medium, and iron-deficient medium supplemented with Fe^3+^ chelator ethylene diamine tetraacetic acid (EDTA) or Fe^2+^ chelator ferrozine. We then quantified the cell wall orientation and wall width using PI staining in the root meristematic zone (Fig. 4, I to K). The disoriented cell walls of *sku5 sks1* roots were significantly restored in the iron-depleted medium, supporting the notion that the wall defect of *sku5 sks1* is caused by excessive iron (Fig. 4, I to K).

Iron-regulated transporter 1 (IRT1) is mainly localized at the outer PM of the root epidermis cells, which delivers Fe^2+^ into root cells from the rhizosphere. Loss-of-function of IRT1 reduces iron uptake (Dubeaux *et al*. 2018). To attenuate the iron content of the *sku5 sks1* mutant in vivo, we generated the *sku5 sks1 irt1* triple mutant (Fig. 4L; Supplemental Fig. S6). The defective cell walls of the *sku5 sks1* double mutant were effectively restored in *sku5 sks1 irt1* triple mutant grown in an iron-sufficient medium (Fig. 4, L to N). To Further analysis by TEM showed that the cell wall protrusions of *sku5 sks1* were also significantly restored in *sku5 sks1 irt1* roots (Fig. 4, O and P). Altogether, these results demonstrated that SKU5 and SKS1 are responsive to iron and play a role in iron homeostasis in the apoplast.

As both ROS and iron levels were elevated in the apoplast of *sku5 sks1* roots, we further aimed to understand whether iron acts upstream of ROS for SKU5/SKS1 regulation or vice versa. We thus detected ROS production of iron chelator-treated *sku5 sks1* roots and iron accumulation of ROS inhibitor (DPI)-treated *sku5 sks1* roots. As a result, iron chelation decreased ROS level, whereas inhibition of ROS generation did not alter iron deposition in the *sku5 sks1* mutant (Supplemental Fig. S7, A to D). Therefore, ROS likely act downstream of iron to participate in cell wall formation in *sku5 sks1* roots.

### GPI anchor is indispensable for SKU5/SKS1 localization and functionality

**Figure 5.**
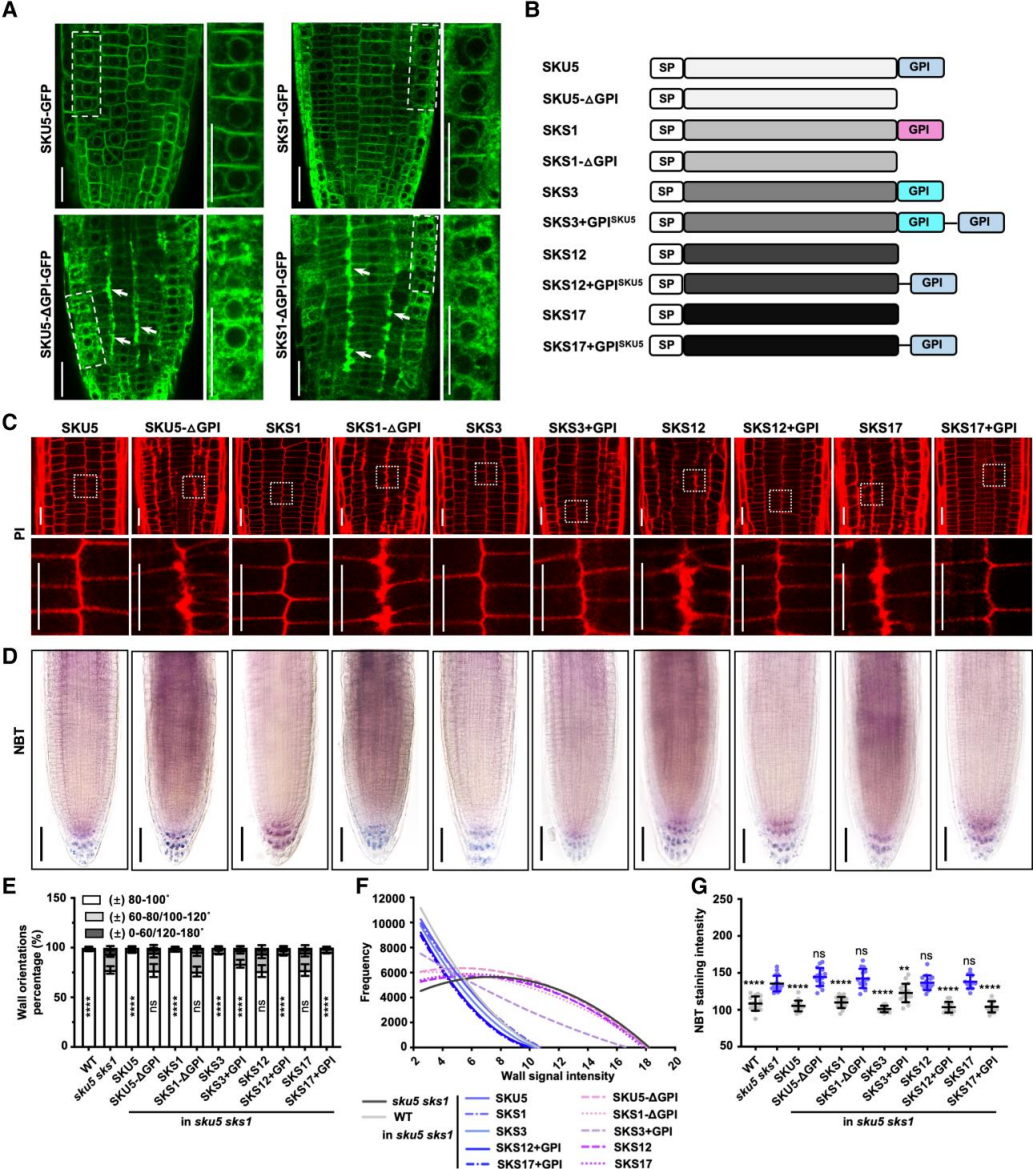
GPI anchor is indispensable for SKU5/SKS1 location and functionality. **A)** Subcellular distribution of full-length and truncated SKU5/SKS1 proteins was visualized in *p35S::SKU5/SKS1-GFP* and *p35S::SKU5/SKS1-ΔGPI-GFP* transgenic plants. The images in the right panels displayed 4× enlarged views of boxed areas in the original images in the left panels. Arrows highlight the secretion of SKU5 and SKS1 to the cell walls along the junction area between the epidermis–cortex and cortex–endodermis cell layers. **B)** Constructs of full-length SKU5, SKS1, SKS3, SKS12, SKS17 proteins, truncated SKU5, SKS1 proteins without GPI motif (SKU/SKS-ΔGPI), as well as modified SKS3, SKS12, SKS17 proteins with additional GPI motif (SKS + GPI^SKU5^). The GPI motif of SKU5 was marked gray, the GPI motif of SKS1 was marked pink, and the GPI motif of SKS3 was marked blue. **C to G)** The generated constructs of truncated- or domain switched-SKS constructs were individually introduced into *sku5 sks1.* Cell morphology (C) and ROS deposition (D) of consequent transgenic plants were stained by PI and NBT. Cell wall orientation (E), PI signal widths signal intensity (F), and ROS signal intensity (G) were quantified. Asterisks indicate significant differences between the column of “± 80–100 ˚”, and the column of *sku5 sks1* is compared to other measurements (E). (E, *n* = 15, 15, 16, 16, 16, 16, 16, 16, 16, 16, 16, and 16; F, *n* = 15 plants for each sample; G, *n* = 16, 16, 16, 15, 18, 14, 15, 16, 15, 15, 14, and 15). Scale bar, 25 µm (A), 20 µm (C), 50 µm (D). Error bar = S.D. *P*-values were determined by two-tailed Student's *t*-test (G) and two-way ANOVA (E) (**P < 0. 01; ****P < 0.0001; ns, not significant).

As GPI-anchored proteins, the potential functions of SKU5 and SKS1 within apoplast prompted us to study the functionality of their GPI tails. Among the 19 SKS proteins, only SKU5, SKS1, and SKS2 contain the classical GPI anchors at the C-terminal region with the amino acids S-561, S-562, and S-563 as the ω site, respectively (Supplemental Fig. S8, A and B). Compared with the PM and endoplasmic reticulum distribution of the intact SKU5 and SKS1, the signal of SKS proteins without GPI tails (termed SKU5/SKS1-ΔGPI) was redistributed in the cytosol (Fig. 5A). Hence, the GPI motif is necessary for the PM association of SKU5 and SKS1.

To further study whether the GPI tail is necessary for SKU5/SKS1-mediated ROS production and cell wall formation, we introduced *p35S::SKU5/SKS1-GFP* and *p35S::SKU5/SKS1-ΔGPI-GFP* in *sku5 sks1* mutant (Supplemental Fig. S9, C and D). The transgenic plants harboring *p35S::SKU5/SKS1-GFP* showed full restoration of the root defects in the *sku5 sks1* mutant (Fig. 5, C to G, Supplemental Fig. S9, A and B). In contrast, *p35S::SKU5/SKS1-ΔGPI-GFP* constructs were unable to rescue the *sku5 sks1* phenotypes (Fig. 5, C to G, Supplemental Fig. S9, A and B).

To further study the importance of the GPI tail, we generated artificial constructs by switching the SKU5 GPI motif in-frame with the tails of 2 non-GPI members, SKS12 and SKS17 (SKS + GPI^SKU5^) (Fig. 5B), and introduced them into *sku5 sks1* mutant. The transgenic lines with comparable expression levels were used for phenotypic analysis (Supplemental Fig. S9, F and G). Overexpression of the native SKS12/SKS17 failed to restore *sku5 sks1* defects, whereas both *p35S::SKS12* + *GPI^SKU5^* and *p35S::SKS17* + *GPI^SKU5^* fully rescued *sku5 sks1* phenotypes (Fig. 5, C to G, Supplemental Fig. S9, A and B). Moreover, SKS3 had a GPI-like motif close to its C-terminal region (Fig. 5B, Supplemental Fig. S8A). Interestingly, both the native SKS3 and SKS3 + GPI^SKU5^ showed restoration of *sku5 sks1* defects, in terms of wall thickening and ROS deposition (Fig. 5, C to G, Supplemental Fig. S9, A, B, and E). Hence, our data unequivocally support that the GPI anchor is indispensable for the functionality of SKU5 and SKS1 proteins.

## Discussion

**Figure 6.**
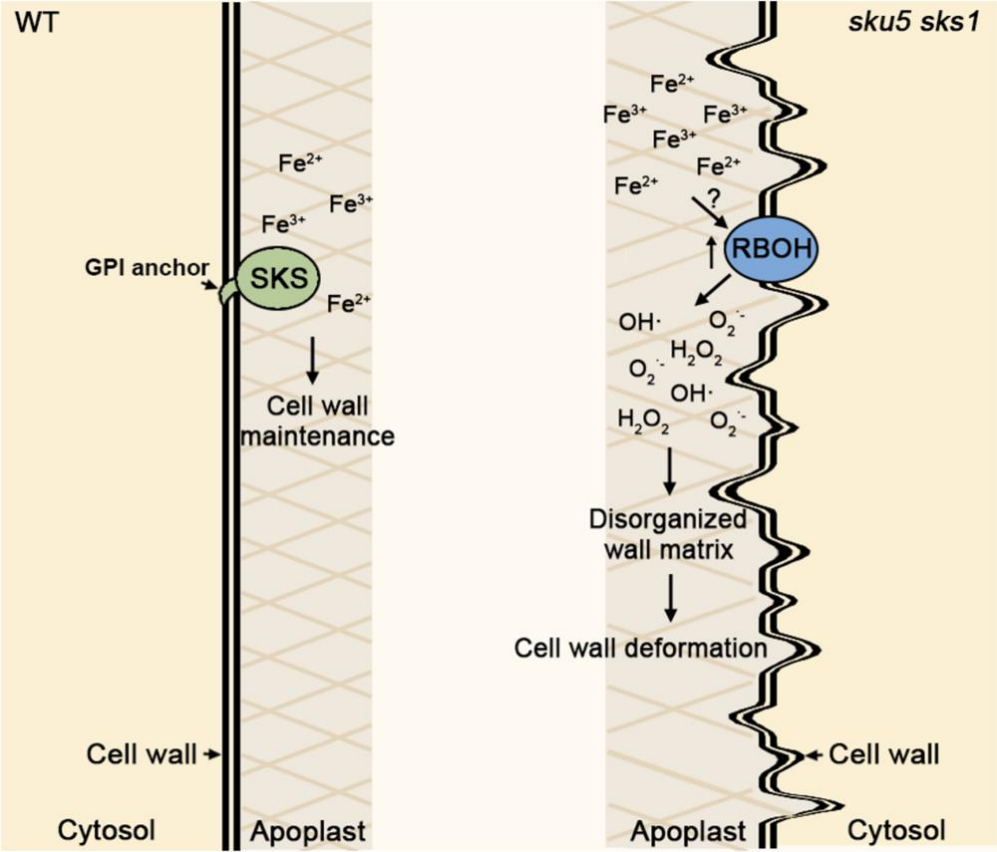
Model of SKU5 and SKS1-coordinated cell wall formation. Speculated model: SKU5 and SKS1 proteins are involved in the regulation of ROS and iron homeostasis in the cell wall, coordinating cell wall formation. Loss-of-function of SKU5 and SKS1 leads to excess iron deposition, elevated RBOH expression, and ROS overproduction in root apoplast, resulting in disorganized wall matrix and cell wall deformation. GPI motif is essential for SKU5 and SKS1 functionality. The “?” indicates the unknown mechanisms that need to be further investigated.

Plant cell walls comprise multiple polysaccharides to provide sufficient mechanical strength and growth expansibility. It is of great importance to stabilize wall nanostructure to maintain cell wall integrity and plasticity. Although ROS-dependent cross-linking of polymers is necessary to sustain wall stability, under certain circumstances, excessive ROS production will destroy the wall matrix, leading to cell wall restructuring. Iron fine-tunes redox-based ROS burst due to the redox cycling of iron between Fe^2+^ and Fe^3+^ forms. The presence of excess iron and ROS in the apoplast has a severe toxic effect, thereby destroying cell wall structure. In this study, we characterized 2 iron-responsive proteins, SKU5 and SKS1, which are involved in the maintenance of cell wall structure through the regulation of ROS levels in the apoplast (Fig. 6). However, the detailed regulatory mechanisms of SKU5 and SKS1 for cell wall formation have not been well clarified in this study. We thus propose the following hypotheses.

### SKU5 and SKS1 may moderate iron homeostasis to regulating the redox state in apoplast

In yeast, the Fet3p-Ftr1p-based iron transport system is important for iron transport across the membrane (Askwith and Kaplan 1997; Kosman 2003). Fet3p is an MCO, which oxidases Fe^2+^ to Fe^3+^ (Hassett *et al*. 1998). Ftr1 is an iron permease that transports Fe^3+^ into cells (Stearman *et al*. 1996). These 2 proteins work together and form a heterodimer to uptake iron (Stearman *et al*. 1996; Kosman 2003). SKU5 is annotated as a structural analog of MCOs, which shares 23% to 27% sequence similarity with AOs and LACs (Sedbrook *et al*. 2002). MCOs comprise a broad class of enzymes responsible for the oxidation of substrates (Hoegger *et al*. 2006); however, the substrate of SKU5 remains unknown. In a redox-based reaction catalyzed by an MCO, electrons from the substrates are accepted at the mononuclear copper center (type I copper-binding center) and are then transferred to the trinuclear copper center (type II/type III copper-binding center) to complete the transportation of 4 electrons (Hoegger *et al*. 2006). However, the ligands for the type I and type III copper-binding center of SKU5 are absent, and none of the SKS proteins contains a central copper motif (Sedbrook *et al*. 2002). A recent study identified a cell wall-localized MCO named LPR1, which has lost the type I copper-binding center but still exhibits ferroxidase function. LPR1 determines the distribution of iron accumulation, ROS deposition, as well as callose production upon phosphate limitation (Muller *et al*. 2015). In our study, we found that the *sku5 sks1* double mutant exhibited a similar phenotype to LPR1 overexpression lines grown in a phosphate-depleted background. This suggests that SKU5 and SKS1 might play a role in redox reactions in the apoplast, and likely act as limiters or inhibitors of a downstream ferroxidase.

Previous studies suggest that excess iron accumulation triggers ROS overproduction, but the underlying molecular mechanism are largely unknown. The available evidences show that the NADPH oxidase inhibitor DPI suppressed iron-dependent ROS accumulation to entirely attenuate ferroptotic cell death in wheat during a pathogen attacking (Dangol *et al*. 2019). In this study, we found that iron was ectopic deposited in the *sku5 sks1* mutant (Fig. 4H). Decreasing the activity of RBOHC which resulted in lower ROS level restored the defective phenotype of *sku5 sks1* roots (Fig. 3J). All these evidences suggest that iron might act upstream or parallelly with RBOH-mediated ROS signaling, involved in SKU5-mediated cell wall formation. Beside of RBOH-mediated ROS production, some of class III peroxidases which are another type of apoplast-localized ROS regulator were activated in *sku5 sks1* mutant on the transcriptional level. Loss-of-function of RBOHC only partially rescued the wall defects of *sku5 sks1*, suggesting the involvement of additional factors. The upregulation of peroxidases implies that peroxidases possibly participate in this process.

### SKU5 and SKS1 might facilitate apoplastic iron transport in Arabidopsis root

Among the essential micronutrients in plants, iron is one of the vital elements required for plant growth. Iron acts as a cofactor for enzymatic reactions and is involved in a wide variety of redox reactions (Kobayashi and Nishizawa 2012; Kobayashi *et al*. 2019; Herlihy *et al*. 2020). Arabidopsis uses reduction-based strategies to uptake iron from the rhizosphere, which is used in rhizosphere acidification, Fe^3+^ chelation, and reduction, as well as Fe^2+^ transported into root epidermal cells (Kobayashi and Nishizawa 2012). After uptake from the rhizosphere, iron is laterally transported from the epidermis to the root xylem, which crosses the cortex and endodermis cells layers, to meet the higher demand for iron for the growth of aerial parts (Wirén *et al*. 1999; Durrett *et al*. 2007; Rellan-Alvarez *et al*. 2010). In this study, we found that loss-of-function of SKU5 and SKS1 results in ectopic iron deposition in the apoplast (Fig. 4, F to H). Iron deposition in the *sku5 sks1* root might have been caused by the obstruction of iron transmembrane transport in the mutant. The PM-localized iron transporter IRT1 has been well-characterized, which mediates the transportation of iron across the membrane from apoplast to cytosol (Zhai *et al*. 2014; Dubeaux *et al*. 2018). IRT1 accumulates at the outer PM domain of epidermis cells, and loss-of-function of IRT1 reduced iron absorption from the rhizosphere, resulting in severe iron deficiency in plants (Vert *et al*. 2002; Dubeaux *et al*. 2018). Nevertheless, iron influx transporters in root apoplast of epidermis–cortex and cortex–endodermis layers have not yet been identified. SKU5 is localized in root epidermis and cortical cell layers, and SKU5 activity was elevated by iron stimulation (Fig. 4C). The abnormal iron accumulation in *sku5 sks1* double mutant, which was restored in the *sku5 sks1 irt1* triple mutant, suggests that SKU5 may be involved in the regulation of apoplastic iron transport in Arabidopsis root.

### GPI motif-dependent membrane association is crucial for the function of SKU5 and SKS1

As an MCO, the ferroxidase-containing domain of FET3 associates with the membrane from the outer leaflet, executing its function of iron oxidation (De Silva *et al*. 1995). SKU5 and SKS1 are GPI-anchored proteins, which also anchor to the PM from the outer leaflet of the membrane (Yeats *et al*. 2018). Some GPI-anchored proteins have been characterized to serve as crucial components during cell wall biosynthesis. For example, LORELEI-like GPI-anchored proteins 2/3 (LLG2/3) are crucial for the activation of ROS production in promoting pollen tube growth (Feng *et al*. 2019), COBRA-like family member COBRA-like 2 mediates crystalline cellulose deposition into the cell wall (Ben-Tov *et al*. 2015), ZERZAUST serves as an atypical β−1,3 glucanase to influence cell wall composition (Vaddepalli *et al*. 2017), and powdery mildew resistant 6 is a pectate lyase-like protein that degrades pectin (Vogel *et al*. 2002). The GPI motif is indispensable for the functionality of all of these proteins. GPI anchor undergoes dynamic modifications, including inositol-deacylation, elimination of ethanolamine phosphate, and fatty acid remodeling, before the GPI-anchored protein is transported to the cell surface (Kinoshita 2015). Such modification is critical for the sorting of GPI-anchored proteins during protein secretion and lateral heterogeneity at the PM (Kinoshita 2015). A previous study showed that GPI anchor serves as a sort signal for plasmodesmata (PD)-related GPI-anchored proteins (Zavaliev *et al*. 2016). GPI signal is sufficient for PD targeting of the PD-related proteins PD-associated β-1,3-glucanases (BG_pap) and callose-binding protein 1 (Zavaliev *et al*. 2016). These 2 PD-related proteins regulate PD permeability via modulating cell wall component callose around the neck region of PD (Levy *et al*. 2007; Simpson *et al*. 2009). Therefore, the functionality of the GPI anchor of SKU5 and SKS1 proteins for cell wall formation is worthy of further investigation.

## Materials and methods

### Plant materials and growth conditions

Arabidopsis (*Arabidopsis thaliana*) plants Wassilewskija (Ws) and Columbia-0 (Col-0) was used as background. T-DNA insertion mutants of *sks1* (Flag_521F09), *sks2* (Flag_607D01), *sku5* (Flag_386B03), *rbohc* (SAIL_1275_E08), and *pSKU5::SKU5-GFP* (in *sku5* mutant background) (Sedbrook *et al*. 2002) were used in this study. For the *pSKS1::SKS1-GFP*, the 1867bp *SKS1* promoter was amplified, and GFP was placed in a frame within the *SKS1* gene in a position after the N-terminal cleavable signal sequence.

To create the loss function mutant of *irt1* by using CRISPR/Cas9 technology, 2 guide RNAs (sgRNAs) both targeting IRT1 (TCAACTGCGCCGGAAGAATG and TCTGGTTGGAGGAACGAAAC) were inserted into the vector pHEE401, the details of vector construction was previously described (Wang *et al*. 2015). The primers used are listed in Supplemental Table S2.

Seeds of *Arabidopsis thaliana* were sown on 1/2 Murashige and Skoog (1/2MS) media with 0.8% w/v agar or 0.8% w/v agarose was used as the standard iron-sufficient (+Fe [50 μM Fe^3+^-EDTA]) medium. 5-day-old seedlings were used for all the experiment, if not specially stated. The iron-deficient medium (–Fe [0 μM Fe^3+^-EDTA]) was prepared from 1/2MS medium by omitting Fe^3+^-EDTA. For medium of different iron concentration, certain concertation of Fe^3+^-EDTA supplied based on -Fe medium. Seeds were sterilized before they were placed on plates. Plants were growth in plates in the growth chamber at 22 °C under 16 h light/8 h dark photoperiod.

### Confocal microscopy observation

Five-day-old seedlings were mounted on 0.8% agar/agarose 1/2MS chamber slides. Images were taken by Leica SP8 or Andor spinning disc confocal microscopes. The settings of laser, intensity, detector, gain, excitation, and detection were GFP: argon, 8%, hybrid detectors (HyD), 100%, 488 nm, 505–550 nm; FM4-64: argon, 20%, HyD, 100%, 514 nm, 570–616 nm; PI: argon, 20%, photomultipliers, 800 v, 514 nm, 570–700 nm; Aniline blue: diode 405, 5%, HyD, 100%, 405 nm, 430–500 nm; OxyBURST Green H2HFF BSA: argon, 5%, HyD, 100%, 488 nm, 500–550 nm; anti-β-1,3-glucan immunostaining: white light laser, 2%, HyD, 60%, 561 nm, 565–650 nm (Sauer *et al*. 2006). All the images in a single experiment were captured with the same settings. The fluorescence signal was quantified by Fiji software (https://fiji.sc) (Schindelin *et al*. 2012).

### TEM microscopy

For TEM observation, root tips of 5-day-old seedlings were excised and prepared with the high pressure freezing (HPF) procedure. The samples were quickly picked into a type B specimen carrier (200-μm well) with 0.15 M sucrose in phosphate buffer (PB buffer) and given the top hat (flat), place the 2 carriers firmly together, and load this sandwich into the sample holder for HPF (Compact-03, Switzerland). Following HPF, the fast-frozen samples were immersed into a freezing tube containing 1% osmium tetroxide in 100% acetone and placed into the freeze substitution (FS) device (Leica EM AFS2, Germany) with the following parameters: T1 = −90 °C for 72 h, S1 = 3 °C/h, T2 = −60 °C for 12 h, S2 = 3 °C/h, T3 = −30 °C for 12 h, then slowly warmed to 4 °C (5 °C/h). Following FS, 3 rinses in 100% acetone at 4 °C, and 1 rinse at room temperature (rt), 15 min each. Next, have were stained in 0.5% Uranyl Acetate dissolved in 90% acetone/10% methanol (filtered before use), for 2 h in the dark at RT. After staining, samples were rinsed 4 times in 100% acetone, 15 min each, at RT, then transferred samples into new 2 ml Eppendorf tubes. After that, Samples were infiltrated in graded mixture (1:3, 1:1, 3:1) of and Spurr's resin (10 g 3,4-epoxycyclohexylmethyl 3,4-epoxycyclohexanecarboxylate, 8 g diglycidyl ether of polypropylene glycol 736, and 25 g nonenyl succinic anhydride), then changed 100% resin 2 times for 4 d on rotator. Finally, samples were embedded in pure resin with 0.7% Dimethylaminoethanol and polymerized for 12 h at 45 °C, and 48 h at 60 °C. The ultrathin sections were sectioned with a microtome (Leica EM UC6), approximately 70 nm, and examined by a transmission electron microscope (FEI Tecnai Spirit 120 kV).

### AFM microscopy

To probe cellulose microfibrils in cell walls, the primary roots of wild-type and mutant seedlings were subjected to AFM as described previously (Zhang *et al*. 2019). The root tips were cut and treated in a peracetic acid solution (11%, v/v) at 85 °C for 3 h. After extensive rinsing, the exposed cell walls of detached root cortex cells were imaged in the air by using a multimode scanning probe microscope (MM-SPM; Bruker) with an advanced NanoScope V Controller (Veeco). All obtained images were scanned in 1-μm scale at 512 × 512 pixels using a ScanAsyst-Air probe (Bruker). The raw images were flattened to remove tilt or bow and then exported in the TIFF format using Nanoscope Analysis (version 1.8; Bruker).

### Imaging of ROS accumulation

Extracellular release of ROS was monitored using OxyBURST Green H2HFF BSA by previously described methods (Monshausen *et al*. 2009; Evans *et al*. 2016). Briefly, 100 μg/mL OxyBURST Green H2HFF BSA dissolved in 1/2 MS and added to a microscope slide for observation by confocal microscopy, as described above. Then, roots from 5-day-old seedlings were placed on a microscope slide, the images were taken starting after 1 min at 20 s intervals for 140 s subsequently. We normalized values by determining the fluorescence intensity of the image at “0 s”, and calculating the difference between all values with the values at “0 s” in the measurement.

The distribution of O_2_^·−^ was determined in the root tip by NBT according to the method as described (Muller *et al*. 2015). Briefly, for O_2_^·−^ detection, 0.5 mg/mL NBT was applied to incubate seedlings for 30 min in 100 mM Na-PB buffer (pH 7.2), and seedlings were optically cleared with chloral hydrate solution (Tyburski *et al*. 2009).

### Callose observation

Callose staining was performed by staining the 5-day-old roots with 1% aniline blue solution containing 50 mM K_3_PO_4%_ and 0.1% Silwet L-77 for 30 min. For β-1,3-glucan immunostaining, roots were captured from 5-day-old seedlings. The protocol referred to the previous method (Sauer *et al*. 2006). β-1,3-glucan antibody (Biosupplies, 1:100) was used as the primary antibody. Alexa Fluor 546 goat antimouse (Life Technologies, 1:500) was used as the secondary antibody.

### Determination of NADPH oxidase activity

The NADPH oxidase activity was determined as previously described with minor modification (Jiang and Zhang 2002; Kaundal *et al*. 2012). For protein extraction and separation of membrane faction, 0.1 g roots were ground into a fine powder and homogenized in 4 volumes of extraction buffer containing 0.5 M sucrose, 50 mM Tris–HCl (pH 7.5), 1 mM EDTA, 100 mM MgCl_2_, 5 mM ascorbic acid sodium, and 1 mM phenylmethylsulfonyl fluoride (PMSF). The homogenized tissue was centrifuged at 5,000 × *g* for 10 min and obtain the supernatant. Total membrane fractions were separated by centrifuging the supernatant at 50,000 × *g* for 60 min at 4 °C and the pellet was suspended in 40 µL Microsomal buffer containing 25 mM Tris (pH 6.8), 0.5 mM EDTA, 0.1 mM MgCl_2_, 0.33 mM sucrose, 1% glycerol, and 1 mM PMSF. The protein content of the microsomal fraction was determined by the Bradford protein assay kit (Beyotime).

The NADPH-dependent O_2_^·−^ -generating activity was determined by following the reduction of sodium, 3′-[1-[phenylamino-carbonyl]-3,4-tetrazolium]-bis(4-methoxy-6-nitro) benzenesulfonic acid hydrate (XTT) by O_2_^·−^. The assay mixture contains 50 mM Tris–HCl (pH 7.5), 0.5 mM XTT, 100 µM NADPH, and 20 µg of membrane fraction. The reaction was initiated with the addition of NADPH, and XTT reduction was determined at 470 nm. To correct for background levels of activity, we prepare 2 blanks, 1 without NADPH and the other replace membrane fraction with water. Rates of O_2_^·−^ generation were calculated using an extinction coefficient of 2.16 × 10^4^ M^−1^ cm^−1^.

### Apoplastic Fe content determination

The determination of Fe content in apoplast was performed as previously described with minor modification (Bienfait *et al*. 1985; Peng *et al*. 2021). Briefly, 5-day-old roots of WT and *sku5 sks1* were cut and transferred to a 50-mL tube with 0.5 mM CaSO_4_. After 15 min, roots were placed in a 15-mL tube with 3 mL 10 mM 2-(N-Morpholino)ethanesulfonic acid, 0.5 mM Ca (NO_3_)_2_, 1.5 mM 2,2′-bipyridyl (pH5.5) at 25 °C. Nitrogen was bubbled through the solution. After 5 min under nitrogen, 150 µL 250 mM Na_2_S_2_O_4_ was added. The *A_520_* of the solution was followed on 200 µL samples after 10 min. To correct for background levels, the first sample was taken just before the addition of dithionite. The apoplastic Fe content was normalized to root fresh weight.

### Iron histochemical staining assay

Iron staining by Perls/DAB and Turnbull/DAB was performed as previously described with minor modification (Muller *et al*. 2015; Wang *et al*. 2019). For Perls staining, excised roots from the seedlings were incubated in the staining solution of a Perls staining kit (Solarbio) for 30 min. For Turnbull staining, roots were incubated in 4% (v/v) HCl, 4% (v/v) K-ferricyanide for 30 min. After Perls and Turnbull staining, roots were washed and incubated in methanol containing 10 mM Na-azide and 0.3% (v/v) H_2_O_2_ for 1 h. After washing with 100 mM Na-PB buffer (pH 7.4), roots were transferred to DAB solution with the concentration of 0.025%. The reaction was stopped by washing with Na-PB buffer and optically clearing with chloral hydrate solution (1 g/mL, 15% glycerol).

The rest of the methods used for cloning strategy; quenching assay, imaging, and analysis; cellulase content determination, etc., are described in Supplemental Materials and Methods.

### Accession numbers

Sequence data from this article can be found in the GenBank/EMBL data libraries under accession numbers: SKU5 (NM_001203775), SKS1 (NM_118656), RBOHC (NM_124485), IRT1 (NM_118089).

## Supplementary Material

kiad207_Supplementary_Data

## Data Availability

The data that support the findings of this study are available from the corresponding author upon reasonable request.

## Supplemental data

The following materials are available in the online version of this article.

**Supplemental Figure S1.** SKU5 and SKS1 redundantly regulate root development.

**Supplemental Figure S2.** CSC trajectory was disrupted in *sku5 sks1*.

**Supplemental Figure S3.** Apoplastic ROS production was increased in *sku5 sks1*.

**Supplemental Figure S4.** *rbohs* rescue the root length of *sku5 sks1*.

**Supplemental Figure S5.** SKS1 activity is upregulated by iron.

**Supplemental Figure S6.** The CRISPR mutant line of *irt1*.

**Supplemental Figure S7.** ROS might act downstream of iron pathway for SKU5-mediated root growth.

**Supplemental Figure S8.** Transmembrane domain and sequence analysis of SKS members.

**Supplemental Figure S9.** Establishment of independent SKSs transgenic plants.

**Supplemental Materials and Methods.**

**Supplemental Table S1.** Co-expression network of *SKU5* gene.

**Supplemental Table S2.** List of primers used for genotyping and RT-qPCR analysis.

**Supplemental Table S3.** Cloning strategy.
