# Supplementary material for: Multi-copper oxidases SKU5 and SKS1 coordinate cell wall formation using apoplastic redox-based reactions in roots
Source: Plant Physiol. 2023 Apr 3;192(3):2243–60. doi: 10.1093/plphys/kiad207 (PMC10315306; doi:10.1093/plphys/kiad207)
Supplement: kiad207_Supplementary_Data [file kiad207_supplementary_data.pdf]

Supplemental Data for:

Multi-copper oxidases SKU5 and SKS1 coordinate cell wall formation using apoplastic redox-based reactions in Arabidopsis root

Chaofan Chen[1,2], Yi Zhang[1,2], Jianfa Cai[2], Yuting Qiu[2], Lihong Li[2], Chengxu Gao[3], Yiqun Gao[4], Meiyu Ke[1,2], Shengwei Wu[1,2], Chuan Wei[1], Jiaomei Chen[2], Tongda Xu[2], Jiří Friml[5], Junqi Wang[6], Ruixi Li[6], Daiyin Chao[4], Baocai Zhang[3], Xu Chen[2,*] and Zhen Gao[2,*]

[1] College of Life Science and Fujian Provincial Key Laboratory of Haixia Applied Plant Systems Biology, Fujian Agriculture and Forestry University, Fuzhou, Fujian, China

[2] FAFU-UCR Joint Center for Horticultural Biology and Metabolomics, Haixia Institute of Science and Technology, Fujian Agriculture and Forestry University, Fuzhou, Fujian, China

[3] State Key Laboratory of Plant Genomics, Institute of Genetics and Developmental Biology, The Innovative Academy of Seed Design, Chinese Academy of Sciences, Beijing 100101, China

[4] National Key Laboratory of Plant Molecular Genetics, CAS Center for Excellence in Molecular Plant Sciences, Shanghai Institute of Plant Physiology and Ecology, Chinese Academy of Sciences, Shanghai 200032, China.

[5] Institute of Science and Technology Austria (IST Austria), Am Campus 1, 3400 Klosterneuburg, Austria.

[6] Department of Biology, Institute of Plant and Food Science, Southern University of Science and Technology, Shenzhen, Guangdong 518055, China

Corresponding authors: Zhen Gao (gaozhen0695@fafu.edu.cn), Xu Chen (chenxu@fafu.edu.cn)

List of Supplemental Figures (9), Method (1), Table (3):

Supplemental Figure S1. SKU5 and SKS1 redundantly regulate root development.

Supplemental Figure S2. CSC trajectory was disrupted in *sku5 sks1*.

Supplemental Figure S3. Apoplastic ROS production was increased in *sku5 sks1*.

Supplemental Figure S4. *rbohs* rescue the root length of *sku5 sks1*.

Supplemental Figure S5. SKS1 activity is upregulated by iron.

Supplemental Figure S6. The CRISPR mutant line of *irt1*.

Supplemental Figure S7. ROS might act downstream of iron pathway for SKU5-mediated root growth.

Supplemental Figure S8. Transmembrane domain and sequence analysis of SKS members.

Supplemental Figure S9. Establishment of independent SKSs transgenic plants.

Supplemental Materials and Methods.

Supplemental Table S1. Co-expression network of *SKU5* gene.

Supplemental Table S2. List of primers used for genotyping and RT-qPCR analysis.

Supplemental Table S3. Cloning Strategy.

Supplemental Figure S1

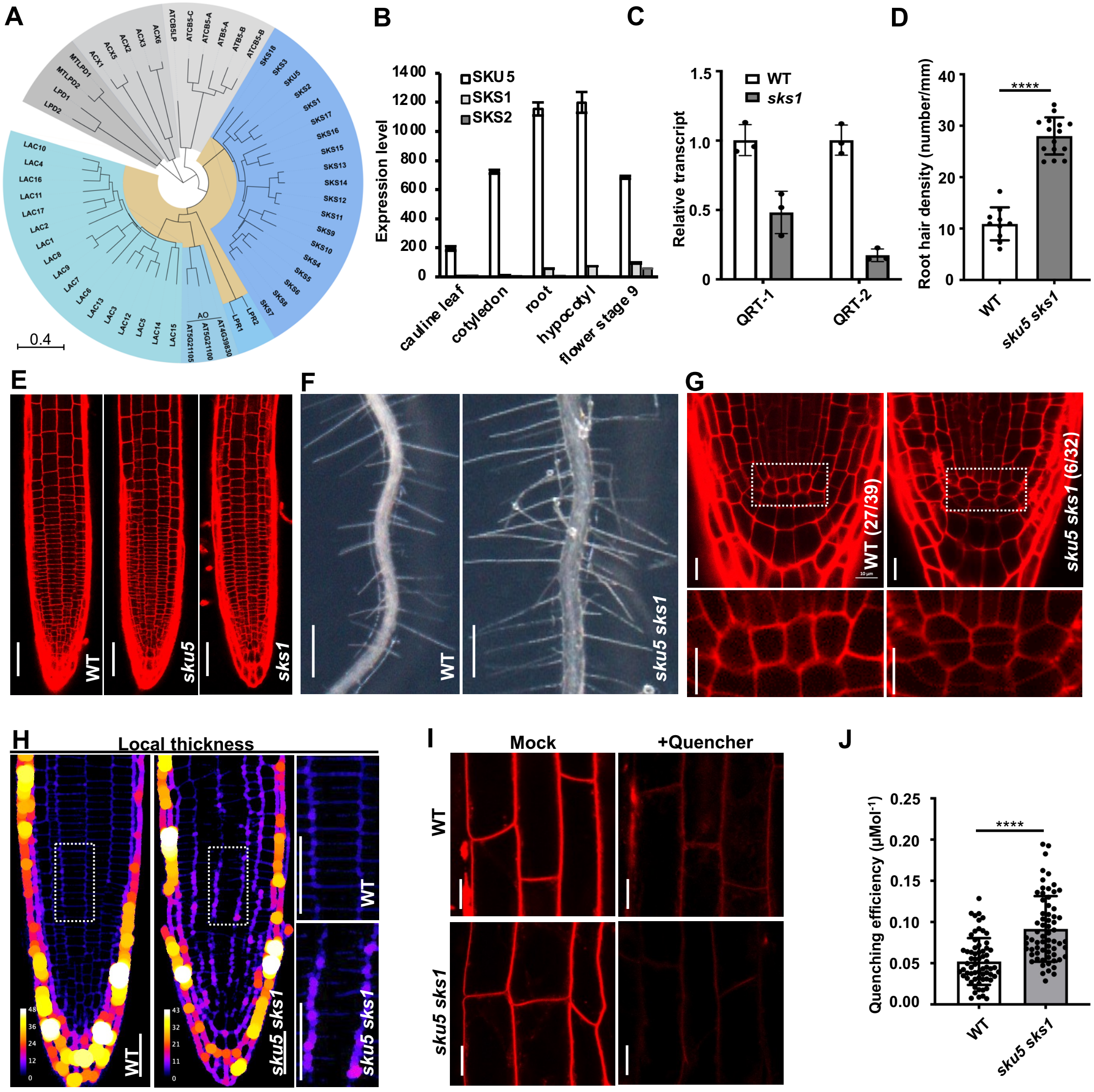

Supplemental Figure S1. SKU5 and SKS1 redundantly regulate root development.

(A) The phylogenetic tree of putative multicopper oxidases (MCOs) from Arabidopsis was constructed using MEGA X. The scale bar represents 0.4 substitution per site.

(B) Expression level of SKU5, SKS1 and SKS2 in different tissues was shown based on the online microarray data.

(C) Transcript level of SKS1 was detected in WT and *sks1* mutant. Two independent primers were used. (n = 3 for each column).

(D, F) Root hairs of 6-day-old WT and *sku5 sks1* roots were observed (F) and quantified as hair density (D) (D, WT, n = 10, *sku5 sks1*, n = 15).

(E) Root cell morphology of WT, *sku5* and *sks1* mutants was observed by PI staining.

(G) Disordered cell growth pattern was observed in the quiescent center (QC) region of *sku5 sks1* roots. Cell morphology around root QC in WT and *sku5 sks1* were observed by PI staining. The images in the bottom panels displayed the 3 × enlarged views of boxed areas from the original images.

(H) Heatmaps of "Local-thickness" in PI-stained WT and *sku5 sks1* roots. Heatmaps were created to profile PI signal width from Figure 1E. Higher signal intensity indicates a larger diameter of PI signal. The images in the right panels displayed 4 × enlarged views of the original images. 80 x 160 pixel region of interest (ROI) was selected to analyze the distribution of PI signal width, which was marked by white frame.

(I-J) *sku5 sks1* roots exhibited larger wall porosity along longitudinal wall direction. WT and *sku5 sks1* root epidermis cells were labeled with FM4-64 and imaged in the absence (Mock) and presence of the trypan blue quenchers (50 µM). (I) Quenching efficiency was quantified in (J) (J, n = 65 for each column).

Scale bar, 50 µm (E), 1 mm (F), 10 µm (G), 20 µm (H) and 10 µm (I).

Error bar = S.D. P-values were determined by two-tailed Student's t-test (D, J) (****p < 0.0001).

Supplemental Figure S2

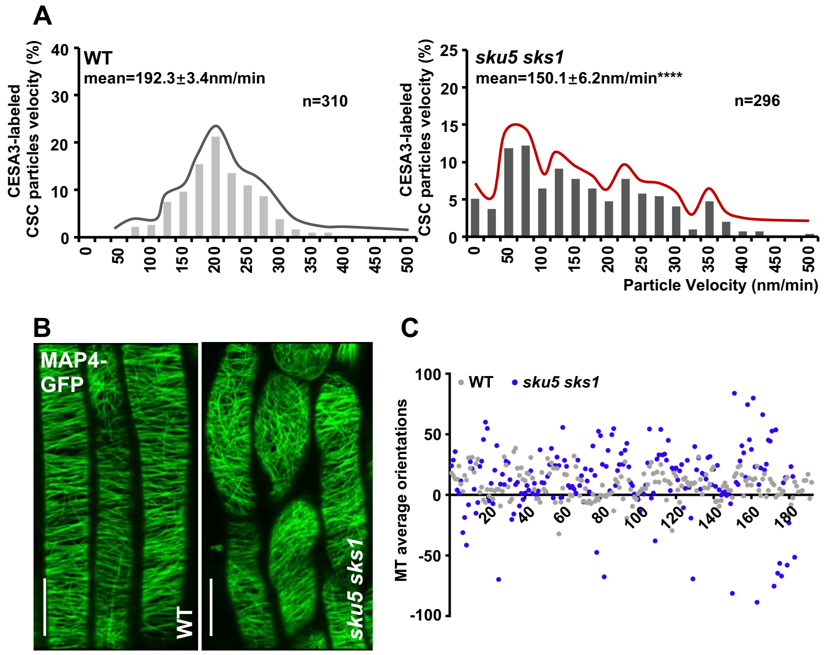

Supplemental Figure S2. CSC trajectory was disrupted in *sku5 sks1*.

(A) Cellulose synthase 3 (CESA3)-labeled cellulose synthase complex (CSC) particles movement velocity was measured in roots of WT and *sku5 sks1*. The grey and red lines indicate distribution trends of CESA3-labeled CSC particles velocity in WT and *sku5 sks1*.

(B-C) Cortical microtubules (MTs) indicated by microtubule-associated protein 4 (MAP4)-GFP were visualized (B) and orientations were quantified (C) in transition zone of WT and *sku5 sks1* roots (C, WT, n=192; *sku5 sks1*, n=183).

Scale bar, 5 μm (B).

Error bar = S.D. P-values were determined by two-tailed Student's t-test (A) (****$p < 0.0001$).

Supplemental Figure S3

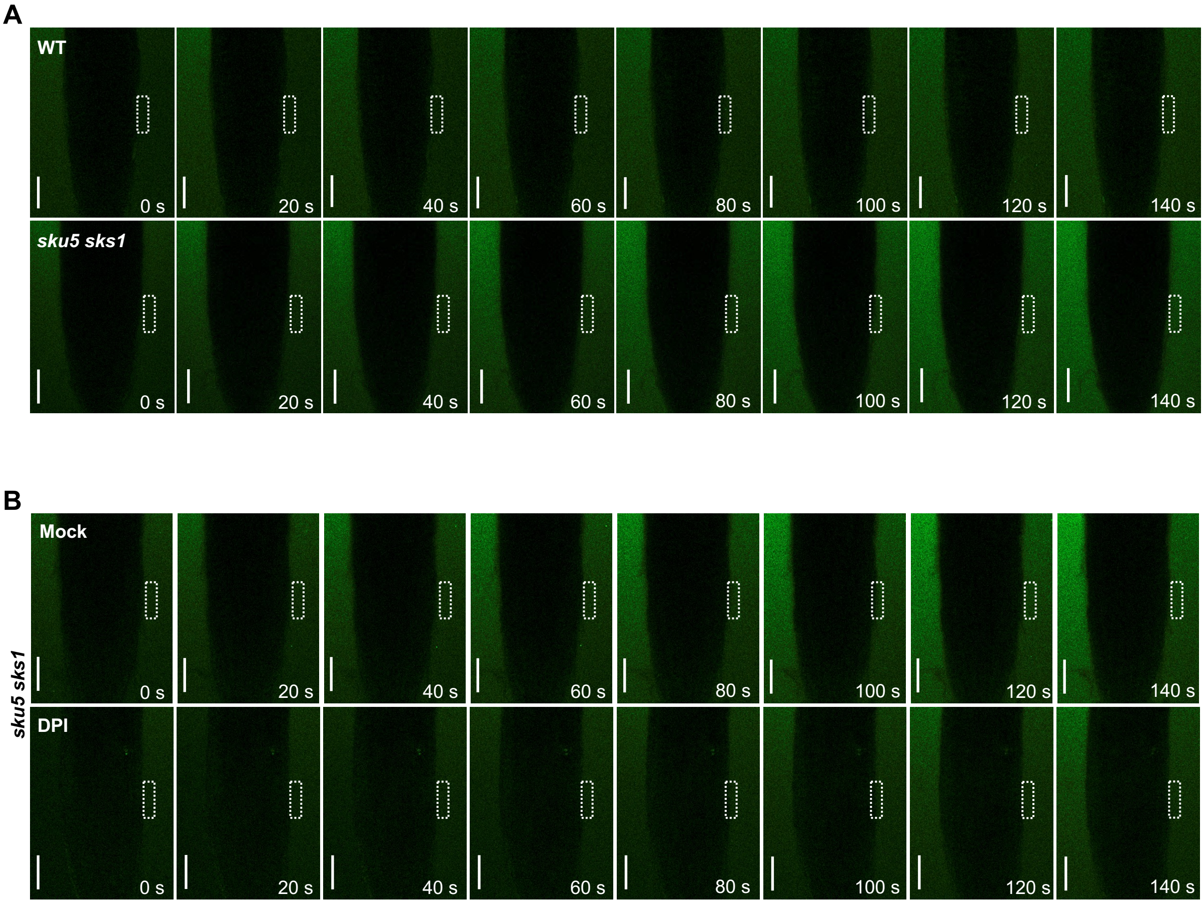

Supplemental Figure S3. Apoplastic ROS production was increased in *sku5 sks1*.
(A) Apoplastic ROS was monitored by OxyBURST Green H2HFF BSA along the cell surface of WT and *sku5 sks1* roots. The images were taken every 20 s for 140 s subsequently. White frame indicated the region of interests (ROIs) which were selected for fluorescence quantification.
(B) Diphenyleneiodonium (DPI) treatment restores the overproduction of apoplastic ROS in *sku5 sks1* roots. Apoplastic ROS was monitored by OxyBURST Green H2HFF BSA along the cell surface of root meristem in mock and DPI (250 nM)-treated *sku5 sks1 roots*. The images were taken every 20 s for 140 s subsequently. White frame indicated the ROIs which were selected for fluorescence quantification.
Scale bar, 50 µm (A) and 50 µm (B).

Supplemental Figure S4

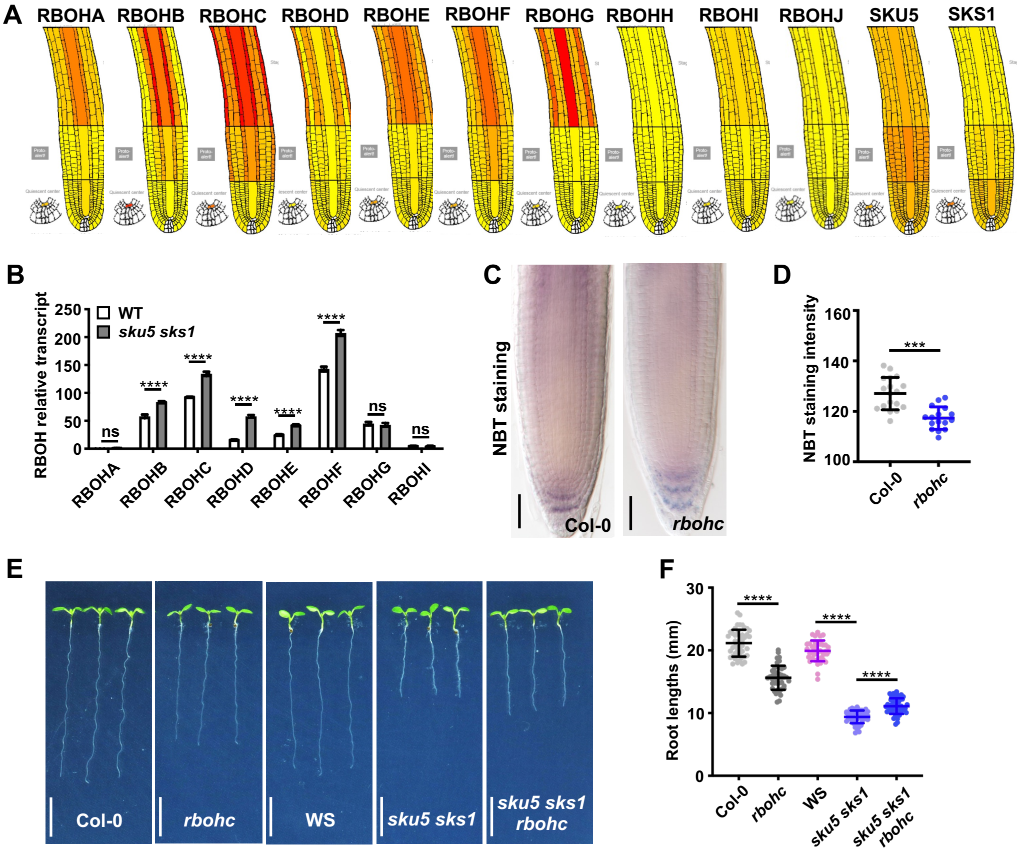

Supplemental Figure S4. *rbohs* rescue the root length of *sku5 sks1*.

(A) Gene expression pattern of RBOHs and SKSs was visualized in Arabidopsis eFP Browser.

(B) Transcript level of RBOHs was detected in WT and *sku5 sks1* root tip by RT-qPCR (n = 3 for each column).

(C-D) $O_2^{\cdot-}$ level of Col-0 and *rbohc* was observed by Nitroblue tetrazolium (NBT) staining (C) and intensity of NBT staining was quantified in root meristematic zone (D) (D, n = 18 and 16).

(E-F) Roots of 6-day-old Col-0, *rbohc*, WS, *sku5 sks1* and *sku5 sks1 rbohc* seedlings were observed (E), and primary root length were qualified (F) (F, n=45 for each column).

Scale bar, 50 μm (C), 6 mm (E).

Error bar = S.D. P-values were determined by two-tailed Student's t-test (B, D, F) (***p < 0.001; ****p < 0.0001, ns, not significant).

Supplemental Figure S5

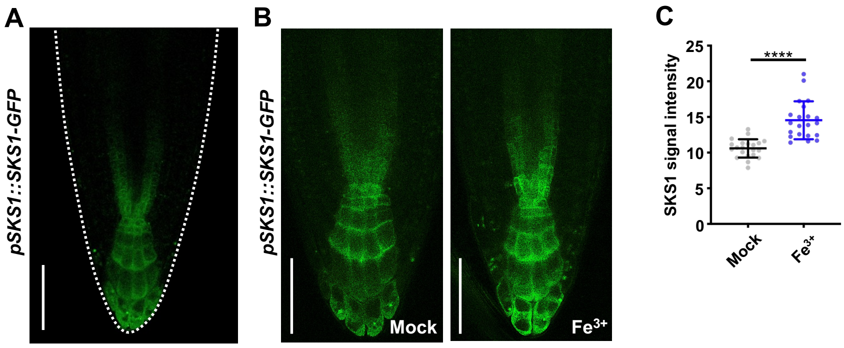

Supplemental Figure S5. SKS1 activity is upregulated by iron.
(A) Subcellular distribution of SKS1 protein was visualized in *pSKS1::SKS1-GFP* roots.
(B-C) Exogenous application of iron promoted SKS1 expression. *pSKS1::SKS1-GFP* was treated with excessive Fe^{3+}-EDTA (300 µM) (B), and total SKS1-GFP signal in root columella stem cells was quantified (C) (C, n = 21 for each column).
Scale bar, 50 µm (A) and 50 µm (B).
Error bar = S.D. P-values were determined by two-tailed Student's t-test (****$p < 0.0001$; ns, not significant).

Supplemental Figure S6

gDNA sequence

IRT1 ACTTCAACTGCGCCGGAAGAATGTGGAAGCG

irt1 ACTTCAACTGCGCCGGAAGAAATGTGGAAGCG (+1bp)

Supplemental Figure S6. The CRISPR mutant line of *irt1*.
irt1 mutant was generated via CRISPR-Cas9 gene-editing approach. The sequence of *IRT1* targeted by the sgRNA was designed in the first exon of *IRT1* gene. sgRNA targeted sequences are framed in green, and the additional base is labeled in red.

Supplemental Figure S7

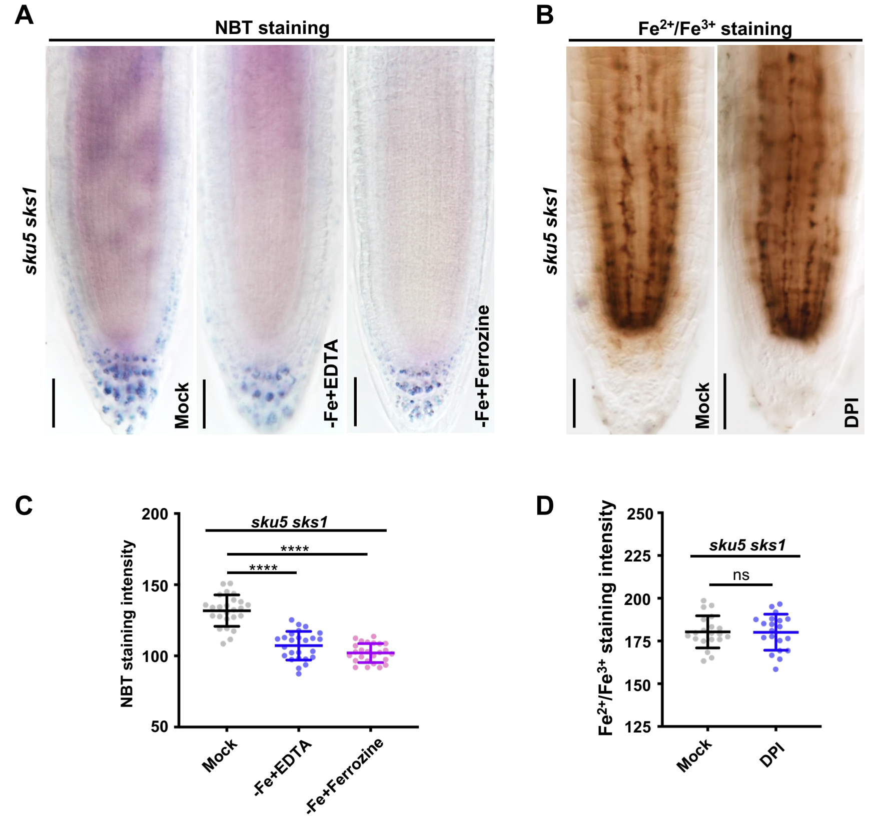

Supplemental Figure S7. ROS might act downstream of iron pathway for SKU5-mediated root growth.

(A, C) $O_2^{\cdot -}$ accumulation in root meristem of *sku5 sks1* which were grown in the iron-sufficient or iron-deficient medium supplied with 300 μM EDTA or 300 μM Ferrozine was visualized by nitroblue tetrazolium (NBT) staining (A). NBT staining signal was quantified in root meristematic zone (C) (C, n = 25 for each column).

(B, D) Iron deposition remained unchanged in diphenyleneiodonium (DPI) -treated *sku5 sks1* roots. Perls/DAB staining was used to observe Fe^{3+} and Fe^{2+} distribution in mock and DPI (250 nM)-treated *sku5 sks1* (B). Signal intensity along longitudinal walls was qualified (D) (D, n = 20 for each column).

Scale bar, 50 μm (A) and 50 μm (B).

Error bar = S.D. P-values were determined by two-tailed Student's t-test (C, D) (****p < 0.0001; ns, not significant).

Supplemental Figure S8

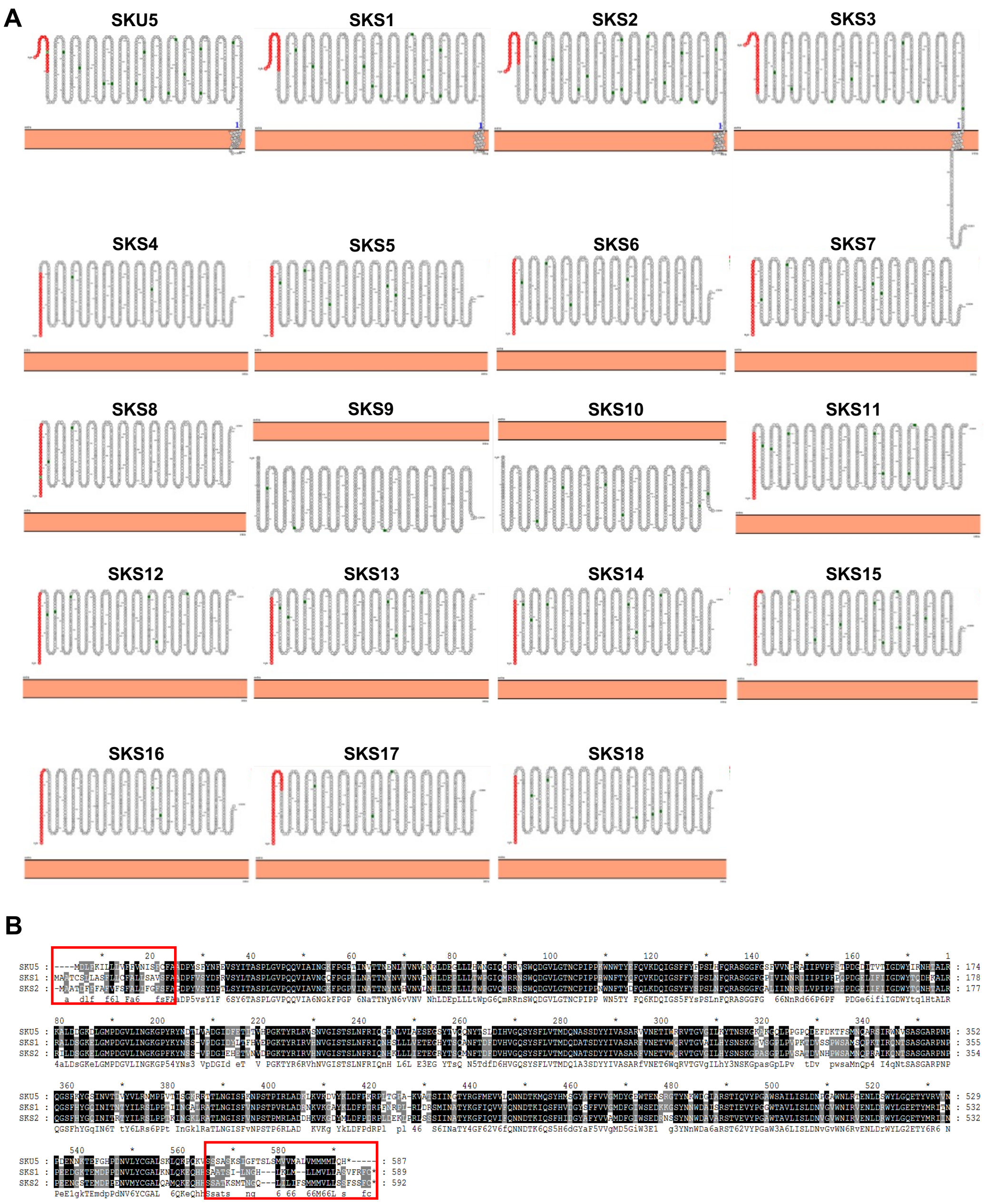

Supplemental Figure S8. Transmembrane domain and sequence analysis of SKS members.

(A) Transmembrane domain was analyzed in SKSs proteins. “1” indicated the transmembrane domain. The green residues indicate N-glycol motif, red color marks the signal peptides, and orange boxes represent plasma membrane (PM).

(B) Sequence alignment of SKU5, SKS1 and SKS2 amino acids. Red frames highlight cleavable signal sequence in the N-terminal and Glycosyl phosphatidylinositol-anchor (GPI)-anchored domains in the C-terminal.

Supplemental Figure S9

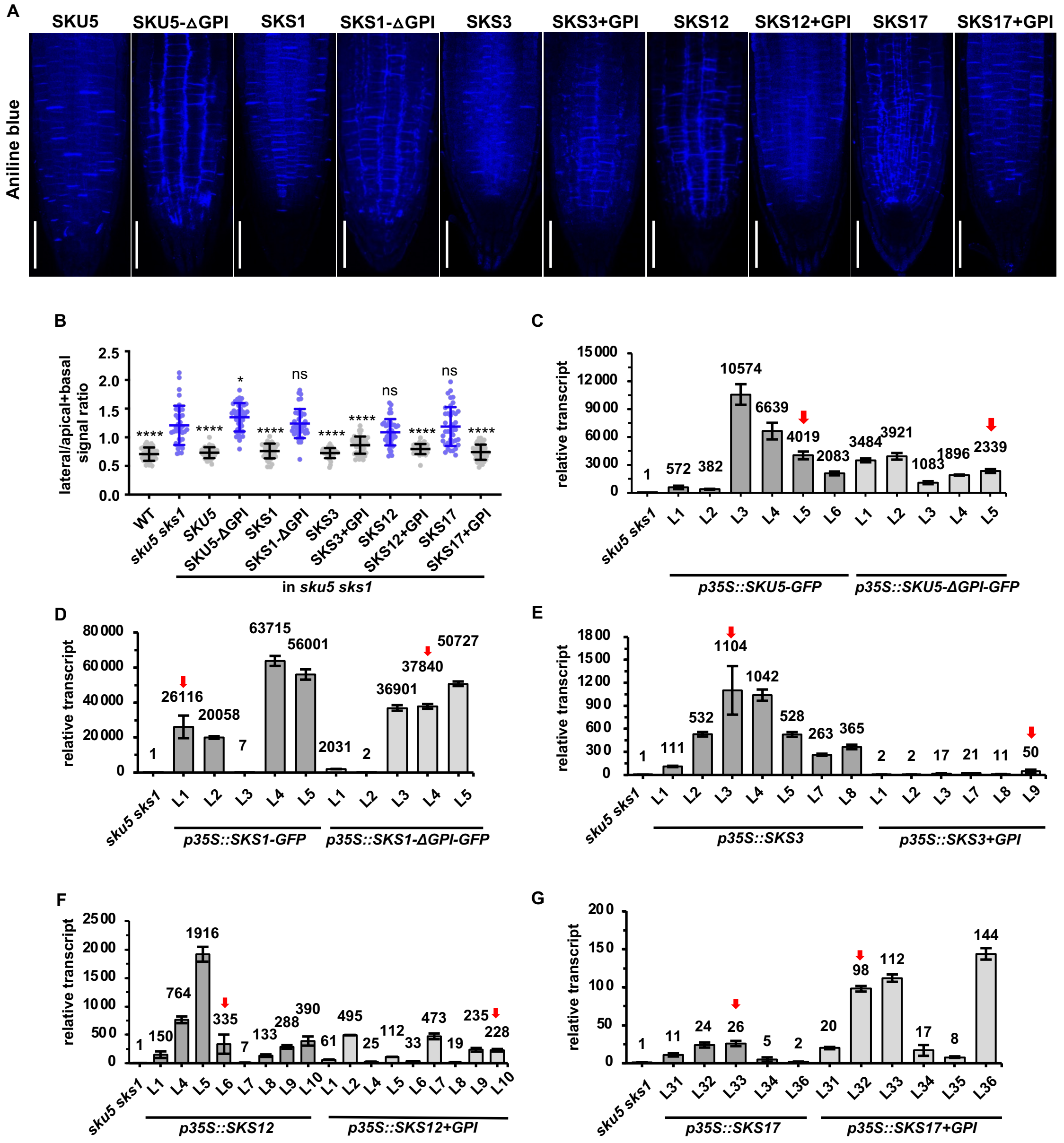

Supplemental Figure S9. Establishment of independent SKSs transgenic plants.

(A-B) Callose deposition of the transgenic SKSs roots was stained by aniline blue (A), and polarity of aniline blue-stained callose was quantified as the signal intensity ratio of lateral divided apical + basal signal (B) (B, n = 40, 40, 40, 40, 40, 40, 38, 40, 40, 33, 40 and 40).

(C-G) Transcript level of SKSs were examined by RT-qPCR in the individual *p35S::SKS* lines (full-length or modified SKS proteins) which were introduced in *sku5 sks1* mutant background. Red arrows marked the represented lines which were used for further phenotype analysis (C-G, n = 3 for each column).

Scale bar, 50 μm (A).

Error bar = S.D. P-values were determined by two-tailed Student's t-test (B) (*p < 0.05; ****p < 0.0001; ns, not significant).

Supplemental Materials and Methods

Cloning strategy

The primers used for genotyping, cloning and RT-qPCR are listed in the Supplemental Table 2. The gateway vectors used for cloning and all the cloning strategies are listed in Supplemental Table 3. Gateway® cloning technology and in-fusion cloning methods were used for generating constructs.

Quenching assay, imaging and analysis

Quenching assay of Arabidopsis root was performed as previously described with minor modification (Liu *et al.*, 2019). Briefly, roots were incubated with 5 μM FM4-64 for 10 min in dark at 4°C and immediately to observe. Quencher (Trypan blue) was dissolved in phosphate buffer (pH 5.8) to reach the concentration of 50 μM, then added to microscope slide to execute observation. After quencher added, samples were imaged immediately using confocal microscope. Quenching efficiency was calculated according to the Stern-Volmer equation (Liu *et al.*, 2019).

Cellulose content determination

Crystalline cellulose content was measured by previously described methods (Foster *et al.*, 2010), with minor modifications. Briefly, 7-day-old *Arabidopsis* roots of the indicated genotypes were transferred to a 2 mL screw-cap Sarstedt tube and serially washed two times with 70% (vol/vol) ethanol, adding with 1:1 chloroform/methanol to resuspend the pellet. Repeat 2-3 times until the pellets become white. 150 μL of acetone was added to each sample, blow down in chemical hood until dry (above steps are the preparation of Alcohol insoluble material (AIR)). Resuspended pellets were dissolved in NaOAc buffer (pH 5.0, [0.1 M]), then heating for 20 minutes at 80°C. Suspension was cooled on ice and reacted with the following buffer at 37°C overnight: 10 μL of 0.01% Sodiumazide (NaN3)); 10 μL Amylase (50 μg/mL); 5 μL Pullulanase. 2 M trifluoroacetic acid (TFA) was added to the air-dried samples, and the final dry pellet was measured by anthrone assay.

RNA-seq, expression annotation and GO enrichment analyses

Arabidopsis seedlings were grown on 1/2 MS medium for 7 days, and primary root were collected. Each sample was collected in triplicates and ground into fine powder with

liquid nitrogen for RNA extraction. The total RNA of the roots was extracted using the TransZol up Plus RNA kit (TransGen Biotech). Deep sequencing of mRNA was performed using illumine nova-seq. 6000 (No-vogene, Beijing, China). To quantify the expression levels of genes, trinity reference was annotated, and paired-end reads were mapped to the assembled transcripts and assigned to genes using RSEM V1.2.20. Differential expression analysis was performed using R package EdgeR V3.32.0. The CPM (Counts per million) of each gene was calculated. Genes with |Log2FC (fold change) |≥1 and FDR≤0.05 were assigned as differentially expressed genes (DEGs). The DEGs were mapped to GO terms in the GO database (http://www. geneontology.org/) in order to calculate the gene numbers for every term. Statistical enrichment of the DEGs for the GO terms implemented by the agriGO v2.0. Significantly enriched GO terms (corrected $p < 0.05$) were identified based hypergeometric test (Du *et al.*, 2010; Tian *et al.*, 2017).

RNA extraction and RT-qPCR analysis

RNA was extracted using the TransZol up Plus RNA kit (TransGen Biotech), and first-strand cDNA was synthesized using Plus All-in-one 1st Strand cDNA Synthesis SuperMix (novoprotein). RT-qPCR was performed using a Bio-Rad CFX96 real-time system (Bio-Rad) with reaction solution containing reverse transcription product, specific primers, ddH_2O and 2 × SYBR qPCR SuperMix Plus (novoprotein). Relative expression level of each gene was calculated using the $2^{-\Delta\Delta CT}$ method.

Histochemical GUS staining

GUS staining solution contains 50 mM sodium phosphate buffer (pH 7.0), 0.1% (v/v) Triton X-100, 0.1 mM $K_3Fe(CN)_6$, 0.1 mM $K_4[Fe(CN)_6]\cdot 3H_2O$, 1 mg/mL X-Gluc, and 1% (v/v) dimethylformamide. Primary roots were stained for 1 h at 37°C, and were optically cleared with chloral hydrate solution before observation.

Western blot analysis

For western blot analysis, total proteins were extracted from 100 mg roots and tested by SDS-PAGE gel. Samples were immunoblotted with SKU5 first antibodies (1:1,000) and the secondary antibody: ECL anti-Rabbit IgG (GE healthcare) (1:10,000). SKU5 antibody was performed as previously described (Sedbrook *et al.*, 2002). Images were taken by Amersham Imager 600.

Plasmolysis

A fresh plasmolysis solution was prepared as following: 3% Macerozyme, 0.4 M D-mannitol, 20 mM MES monohydrate and 10 mM Tris-HCL (PH 7.5). We adjusted the PH to 5.8 for stable GFP observation. The solution was firstly warmed up for 10 minutes at 55 °C, then cooled down at room temperature.

Cell biological quantification methods.

i. Quantification of cell wall orientations: 5-day-old seedlings were stained by PI, and pictures of root tips were taken by Leica SP8. The angle of the cortex and epidermal cell walls which is perpendicular to the axis of root growth was measured in the root meristematic zone by Fiji. Root growth axis was defined as 0°/(±）180° and the deviated angels were grouped into three categories: (±) 80°-100°, (±) 60°-80°/100°-120°, (±) 0°-60°/120°-180° (according to the schematic diagram in Figure 1H). The proportion of deviated angles was also calculated.

ii. Quantification of PI signal width: “Local thickness” in Fiji plug-in was incorporated to create a heatmap profiling of PI signal, which has been applied to evaluate wall/membrane thickness (Dougherty & Kunzelmann, 2007; Rothschild *et al.*, 2017; Nabuqi *et al.*, 2020). After picture transformation by “local thickness” plug-in, the higher signal intensity indicates a larger diameter of wall/membrane (Supplemental Figure1H). Further, distribution of PI-labelled wall thickness can be automatically tracked as profile.

iii. CESA3 trajectories assay: For CSC particle orientations and velocity quantification, the trajectory of CESA3-GFP was taken in total 10 min by Andor spinning disk confocal microscope with following sittings: 800 ms exposure and 10 s interval for each picture. The recorded time-lapse movies were corrected for drift and bleaching using the Fiji plug-ins “stackreg,” “subtract background,” “enhance contract” and “bleach correction” with default settings. In cases of weak intensity, we further applied the “average intensity” plug-in. Velocities were analyzed using the “kymograph” in Fiji software. More than 100 kymographs were analyzed per cell. The dashed yellow line indicates the slope of a representative CESA-containing particle (Sanchez-Rodriguez *et al.*, 2017). For CSC trajectories length quantification, time-averaged projections of CSC trajectories were formed, and the length of CSC trajectories were measured.

iv. Quantification of average microtubule orientations: “FibriTool” in Fiji plug-in was based on the concept of nematic tensor, which can provide a quantitative description of the average orientation of MAP4-GFP in cells (Boudaoud *et al.*, 2014).

v. Quantification of callose and glucan staining polarity: the fluorescence intensity in apical, basal, left lateral and right lateral sides of every cell in root meristematic zone were individually measured by Fiji. The polarity was quantified as the signal ratio of (left lateral + right lateral)/ (apical + basal) signal.

vi. Quantification of NBT staining intensity: NBT staining pictures were taken by NIKON-U with DIC. Next, the picture color was inverted, the staining intensity of root meristematic zone were measured by Fiji.

vii. Quantification of fluorescence signal intensity of *pSKU5::SKU5-GFP*: The pictures were applied with "subtract background" to reduce background noise. Next, the signal intensity was measured in whole root meristem region by Fiji.

viii. Quantification of Turnbull/DAB and Perls/DAB staining intensity: the staining intensity of root QC region and longitudinal signal were individually measured by Fiji on DIC pictures which are inverted colors.

Phylogenetics analysis and protein domain prediction

Arabidopsis thaliana putative MCOs genes were obtained from TAIR (https://www.arabidopsis.org). Protein sequences of these genes aligned with Clustal X (Larkin *et al.*, 2007). A phylogenetic tree was constructed using MEGA X (Kumar *et al.*, 2018) with neighbor-joining (NJ) criteria and verified using the maximum likelihood (ML). A bootstrap test (1,000 replicates) was conducted based on multiple alignments of proteins sequence of MCOs genes. Co-expression prediction was performed on STRING (https://string-db.org) (Szklarczyk *et al.*, 2021). The transmembrane domain analysis was constructed by PROTTER (http://wlab.ethz.ch/protter/start/) (Omasits *et al.*, 2014). For GPI anchor prediction, the website (http://gpcr.biocomp.unibo.it/predgpi/pred.htm) was used to predict GPI motif in SKS family proteins (Pierleoni *et al.*, 2008). N-terminal cleavable signal sequence was conducted by SignalP version 4.0 (https://services.healthtech.dtu.dk/service.php?SignalP) (Petersen *et al.*, 2011).

Statistical analysis, image analysis, and figure preparation

Statistical data were analyzed in Graphpad Prism 7 (GraphPad Software, La Jolla, California USA, www.graphpad.com) using two-tailed Student's t-test and two-way ANOVA. Statistical images were generated by Graphpad Prism 7. Camera and confocal images were prepared with Fiji (https://fiji.sc). All the experiments were carried out at least in triplicate.

Boudaoud A, Burian A, Borowska-Wykret D, Uyttewaal M, Wrzalik R, Kwiatkowska D, Hamant O. 2014. FibrilTool, an ImageJ plug-in to quantify fibrillar structures in raw microscopy images. *Nat Protoc* **9**(2): 457-463.

Dougherty R, Kunzelmann KH. 2007. Computing Local Thickness of 3D Structures with ImageJ. *Microscopy and Microanalysis* **13**(S02).

Du Z, Zhou X, Ling Y, Zhang Z, Su Z. 2010. agriGO: a GO analysis toolkit for the agricultural community. *Nucleic Acids Res* **38**(Web Server issue): W64-70.

Foster CE, Martin TM, Pauly M. 2010. Comprehensive compositional analysis of plant cell walls (lignocellulosic biomass) part II: carbohydrates. *J Vis Exp*(37).

Kumar S, Stecher G, Li M, Knyaz C, Tamura K. 2018. MEGA X: Molecular Evolutionary Genetics Analysis across Computing Platforms. *Mol Biol Evol* **35**(6): 1547-1549.

Larkin MA, Blackshields G, Brown NP, Chenna R, McGettigan PA, McWilliam H, Valentin F, Wallace IM, Wilm A, Lopez R, et al. 2007. Clustal W and Clustal X version 2.0. *Bioinformatics* **23**(21): 2947-2948.

Liu X, Li J, Zhao H, Liu B, Gunther-Pomorski T, Chen S, Liesche J. 2019. Novel tool to quantify cell wall porosity relates wall structure to cell growth and drug uptake. *J Cell Biol* **218**(4): 1408-1421.

Nabuqi, Nuoendagula, Wu S, Takata N, Sakamoto S, Yamamoto M, Uesugi M, Déjardin A, Pilate G, Taniguchi T, et al. 2020. Simultaneous manipulation of lignin structure and secondary cell wall formation in transgenic poplar. *Journal of Wood Science* **66**(1).

Omasits U, Ahrens CH, Muller S, Wollscheid B. 2014. Protter: interactive protein feature visualization and integration with experimental proteomic data. *Bioinformatics* **30**(6): 884-886.

Petersen TN, Brunak S, von Heijne G, Nielsen H. 2011. SignalP 4.0: discriminating signal peptides from transmembrane regions. *Nat Methods* **8**(10): 785-786.

Pierleoni A, Martelli PL, Casadio R. 2008. PredGPI: a GPI-anchor predictor. *BMC Bioinformatics* **9**: 392.

Rothschild PR, Salah S, Berdugo M, Gelize E, Delaunay K, Naud MC, Klein C, Moulin A, Savoldelli M, Bergin C, et al. 2017. ROCK-1 mediates diabetes-induced retinal pigment epithelial and endothelial cell blebbing: Contribution to diabetic retinopathy. *Sci Rep* **7**(1): 8834.

Sanchez-Rodriguez C, Ketelaar K, Schneider R, Villalobos JA, Somerville CR, Persson S, Wallace IS. 2017. BRASSINOSTEROID INSENSITIVE2 negatively regulates cellulose synthesis in Arabidopsis by phosphorylating cellulose synthase 1. *Proc Natl Acad Sci U S A* **114**(13): 3533-3538.

Sedbrook JC, Carroll KL, Hung KF, Masson PH, Somerville CR. 2002. The Arabidopsis SKU5 gene encodes an extracellular glycosyl phosphatidylinositol-anchored glycoprotein involved in directional root growth. *Plant Cell* **14**(7): 1635-1648.

Szklarczyk D, Gable AL, Nastou KC, Lyon D, Kirsch R, Pyysalo S, Doncheva NT, Legeay M, Fang T, Bork P, et al. 2021. The STRING database in 2021: customizable protein-protein networks, and functional characterization of user-uploaded gene/measurement sets. *Nucleic Acids Res* **49**(D1): D605-D612.

Tian T, Liu Y, Yan H, You Q, Yi X, Du Z, Xu W, Su Z. 2017. agriGO v2.0: a GO analysis toolkit for the agricultural community, 2017 update. *Nucleic Acids Res* **45**(W1): W122-W129.

**Supplemental Table S1. Co-expression network of *SKU5* gene.**		
#node	identifier	annotation
SKS1	AT4G25240.1	SKU5 similar 1
SKS4	AT4G22010.1	SKU5 similar 4
SKU5	AT4G12420.1	Monocopper oxidase-like protein SKU5; May be a monocopper oxidase of unknown specificity. Involved in directional growth processes, possibly by participating in cell wall expansion
ENODL17	AT5G15350.1	Lamin-like protein
FLA2	AT4G12730.1	FASCICLIN-like arabinogalactan 2; May be a cell surface adhesion protein
FLA8	AT2G45470.1	FASCICLIN-like arabinogalactan protein 8; May be a cell surface adhesion protein
HIPL2	AT5G62630.1	HIPL2 protein
SOS5	AT3G46550.1	Salt overly sensitive 5; May be a cell surface adhesion protein that is required for normal cell expansion
SVL1	AT5G55480.1	SHV3-like 1; Involved in primary cell wall organization. Required for the accumulation of crystalline cellulose
WDL1	AT3G04630.1	WVD2-like 1; Microtubule-associated protein (MAP) that regulates the orientation of interphase cortical microtubules. Modulates both rotational polarity and anisotropic cell expansion during organ growth. Promotes clockwise root and etiolated hypocotyls coiling, clockwise leaf curling, but left-handed petiole twisting
WVD2	AT5G28646.1	WAVE-DAMPENED 2; Microtubule-associated protein (MAP) that regulates the orientation of interphase cortical microtubules. Able to bundle microtubules in vitro. Modulates both rotational polarity and anisotropic cell expansion during organ growth. Promotes clockwise root and etiolated hypocotyls coiling, clockwise leaf curling, but left-handed petiole twisting
XTH4	AT2G06850.1	Endoxyloglucan transferase (EXGT-A1) gene; Catalyzes xyloglucan endohydrolysis (XEH) and/or endotransglycosylation (XET). Cleaves and religates xyloglucan polymers, an essential constituent of the primary cell wall, and thereby participates in cell wall construction of growing tissues
COBRA	AT5G60920.1	COBRA; Involved in determining the orientation of cell expansion, probably by playing an important role in cellulose deposition. May act by recruiting cellulose synthesizing complexes to discrete positions on the cell surface
AT4G36750	AT4G36750.1	Quinone reductase family protein; Catalyzes the transfer of electrons from NADH and NADPH to reduce quinone to the hydroquinone state

RBOHA	AT5G07390.1	Respiratory burst oxidase-A; Calcium-dependent NADPH oxidase that generates superoxide
RBOHB	AT1G09090.2	Respiratory burst oxidase-B; Calcium-dependent NADPH oxidase that generates superoxide
RBOHD	AT5G47910.1	Respiratory burst oxidase-D; Calcium-dependent NADPH oxidase that generates superoxide. Involved in the generation of reactive oxygen species (ROS) during incompatible interactions with pathogens and in UV-B and abscisic acid ROS-dependent signaling. Might be required for ROS signal amplification during light stress
RBOHE	AT1G19230.2	Riboflavin synthase-like protein; Calcium-dependent NADPH oxidase that generates superoxide
RBOHF	AT1G64060.1	Respiratory burst oxidase; Calcium-dependent NADPH oxidase that generates superoxide. Generates reactive oxygen species (ROS) during incompatible interactions with pathogens and is important in the regulation of the hypersensitive response (HR). Involved in abscisic acid-induced stomatal closing and in UV-B and abscisic acid ROS-dependent signaling
RBOHG	AT4G25090.1	Riboflavin synthase-like superfamily protein; Calcium-dependent NADPH oxidase that generates superoxide
RBOHH	AT5G60010.1	Putative respiratory burst oxidase-H; Calcium-dependent NADPH oxidase that generates superoxide
RBOHI	AT4G11230.1	Putative respiratory burst oxidase-I; Calcium-dependent NADPH oxidase that generates superoxide
RBOHJ	AT3G45810.1	Putative respiratory burst oxidase-J; Calcium-dependent NADPH oxidase that generates superoxide
RHD2	AT5G51060.1	ROOT HAIR DEFECTIVE 2; Calcium-dependent NADPH oxidase that generates superoxide. Required for H(2)O(2) production in response to K(+) deficiency and for the generation of reactive oxygen species (ROS) that regulate cell expansion through the activation of Ca(2+) channels
FRO1	AT1G01590.1	Ferric reduction oxidase 1; Ferric chelate reductase involved in iron reduction in roots (By similarity). May participate in the transport of electrons to a Fe(3+) ion via FAD and heme intermediates
FRO2	AT1G01580.1	Ferric reduction oxidase 2; Flavocytochrome that transfers electrons across the plasma membrane to reduce ferric iron chelates to form soluble ferrous iron in the rhizosphere. May be involved in the delivery of iron to developing pollen grains. Acts also as a copper-chelate reductase. Involved in glycine betaine-mediated chilling tolerance and reactive oxygen species accumulation
FRO3	AT1G23020.2	Ferric reduction oxidase 3; Ferric chelate reductase involved in iron reduction in roots. May participate in

		the transport of electrons to a Fe(3+) ion via FAD and heme intermediates
FRO4	AT5G23980.1	Ferric reduction oxidase 4; Ferric chelate reductase. May participate in the transport of electrons to a Fe(3+) ion via FAD and heme intermediates
FRO5	AT5G23990.1	Ferric reduction oxidase 5
FRO6	AT5G49730.1	Ferric reduction oxidase 6; Ferric chelate reductase involved in iron uptake by shoot and leaf cells. May participate in the transport of electrons to a Fe(3+) ion via FAD and heme intermediates
FRO7	AT5G49740.1	Ferric reduction oxidase 7; Ferric chelate reductase involved in iron mobilization from the cytosol into the chloroplast. May participate in the transport of electrons to a Fe(3+) ion via FAD and heme intermediates. Might be involved iron homeostasis in trichomes
FRO8	AT5G50160.1	Ferric reduction oxidase 8; Ferric chelate reductase probably involved in iron reduction in leaf veins for transport. May participate in the transport of electrons to a Fe(3+) ion via FAD and heme intermediates
HCC1	AT3G08950.1	Homologue of the copper chaperone SCO1; Thought to play a role in cellular copper homeostasis, mitochondrial redox signaling or insertion of copper into the active site of COX. Plays an essential role in embryo development
HCC2	AT4G39740.1	Homologue of copper chaperone SCO1 2; Thought to play a role in cellular copper homeostasis, mitochondrial redox signaling or insertion of copper into the active site of COX (By similarity). Participates in copper and redox homeostasis
HMA1	AT4G37270.1	Heavy metal atpase 1; Involved in cadmium/zinc transport
HMA2	AT4G30110.1	Heavy metal atpase 2; Plays an important role in zinc transport and homeostasis. Could also be involved in cadmium detoxification
HMA3	AT4G30120.1	Heavy metal atpase 3
HMA4	AT2G19110.1	Heavy metal atpase 4; Involved in cadmium/zinc transport
HMA5	AT1G63440.1	Heavy metal atpase 5; Involved in copper import into the cell. May play a role in copper detoxification in roots
HMA6	AT4G33520.2	P-type ATP-ase 1; Mediates copper transfer across the plastid envelope. Required for the delivery of copper into the plastid stroma, which is essential for the function of copper proteins. Seems to be selective for monovalent copper Cu(+) transport. Plays also a role in glucose signaling-mediated cell proliferation of root meristem in non-green tissues
HMA8	AT5G21930.1	P-type ATPase; Mediates copper transfer across the chloroplast thylakoid membrane. Required for copper

		delivery into the thylakoids lumen, which is essential for the function of copper proteins
NRAMP3	AT2G23150.1	Metal transporter Nramp3; Vacuolar metal transporter involved in intracellular metal homeostasis. Can transport iron (Fe), manganese (Mn) and cadmium (Cd). Regulates metal accumulation under Fe starvation. Acts redundantly with NRAMP4 to mobilize vacuolar Fe and provide sufficient Fe during seed germination. In association with NRAMP4, required for optimal growth and photosynthesis under Mn deficiency. Exports Mn from vacuoles in leaf mesophyll cells, making Mn available for functional photosystem II in chloroplasts. Involved in basal resistance to the bacterial pathogen E.chrysanthemi
RAN1	AT5G44790.1	RESPONSIVE-TO-ANTAGONIST 1; Involved in copper import into the cell. Essential for ethylene signaling, which requires copper. Acts by delivering copper to create functional hormone receptors
TSC10B	AT5G19200.1	TSC10B; Catalyzes the reduction of 3-ketodihydrosphingosine (KDS) to dihydrosphingosine (DHS). Required for sphingolipid biosynthesis. In plants, sphingolipids seems to play a critical role in mineral ion homeostasis, most likely through their involvement in the ion transport functionalities of membrane systems in the root. Is stereospecific for D-erythro-DHS production and does not produce L-threo-DHS
APG2	AT2G01110.1	ALBINO AND PALE GREEN 2; Part of the twin-arginine translocation (Tat) system that transports large folded proteins containing a characteristic twin-arginine motif in their signal peptide across the thylakoid membrane. Involved in delta pH-dependent protein transport required for chloroplast development, especially thylakoid membrane formation. TATC and TATB mediate precursor recognition, whereas TATA facilitates translocation
AT1G29740	AT1G29740.1	Leucine-rich repeat transmembrane protein kinase
AT1G68780	AT1G68780.1	RNI-like superfamily protein
BSK7	AT1G63500.1	Brassinosteroid-signaling kinase 7
GLT1	AT5G53460.1	Glutamate synthase 1 [NADH]; Involved in glutamate biosynthesis. Required for non- photorespiratory ammonium assimilation. Probably involved in primary ammonium assimilation in roots
IMK2	AT3G51740.1	Inflorescence meristem receptor-like kinase 2
PDIL1-6	AT3G16110.1	PDI-like 1-6; Acts as a protein-folding catalyst that interacts with nascent polypeptides to catalyze the formation, isomerization, and reduction or oxidation of disulfide bonds
Each cluster is filled with different color according to Figure 1A.		

Supplemental Table S2. List of primers used for genotyping and RT-qPCR analysis.

Name	Forward primers (F) and Reverse primers (R)
SKU5-full length	F: GGGGACAAGTTTGTACAAAAAAGCAGGCTTTATGGATTTGTTCAAGATCCT<br>R: GGGGACCACTTTGTACAAGAAAGCTGGGTTTCAATGCTGAAGCATCATCAT
SKU5-ΔGPI	F: GGGGACAAGTTTGTACAAAAAAGCAGGCTTTATGGATTTGTTCAAGATCCT<br>R: GGGGACCACTTTGTACAAGAAAGCTGGGTTTGAAACCTTCTGTGGCCTGAAA
SKS1-full length	F: GGGGACAAGTTTGTACAAAAAAGCAGGCTTTATGGCGGCGACTTGT<br>R: GGGGACCACTTTGTACAAGAAAGCTGGGTTTCAGCAAAATCTGAACACCGAGG
SKS1-ΔSP	F: GGGGACAAGTTTGTACAAAAAAGCAGGCTTTGCGGACCCTTTCGTCTCC<br>R: GGGGACCACTTTGTACAAGAAAGCTGGGTTTCAGCAAAATCTGAACACCGAGG
proSKS1+SP	F: GGCCAGTGCCAAGCTTGATAGTTTGTGTAAAATATACTAAGCACTTCCA<br>R: CTCGCCCTTGCTCACCATACTAGTCGCGAAAGAAACGGCG
GFP+SKS1-ΔSP	F: CGCCGTTTCTTTCGCGACTAGTATGGTGAGCAAGGGCGAG<br>R: TATGACCATGTCTAGTCAGCAAAATCTGAACACCGAGG
SKS1-ΔGPI	F: GGGGACAAGTTTGTACAAAAAAGCAGGCTTTATGGCGGCGACTTGT<br>R: GGGGACCACTTTGTACAAGAAAGCTGGGTTGTGGTGTTGTTCCCTGCAAC
SKS3-full length	F: GGGGACAAGTTTGTACAAAAAAGCAGGCTTTATGCGGTGCTTTCCACC<br>R: GGGGACCACTTTGTACAAGAAAGCTGGGTTCTAATATGATATCCGATCCCGGTTTTTTTCG
SKU5-GPI overlap with SKS3	F: CGAAAAAAACCGGGATCGGATATCATATTCGTCGGCATCGAAGAGC<br>R: GCTCTTCGATGCCGACGAATATGATATCCGATCCCGGTTTTTTTCG
SKS3+SKU5-GPI	F: GGGGACAAGTTTGTACAAAAAAGCAGGCTTTATGCGGTGCTTTCCACC<br>R: GGGGACCACTTTGTACAAGAAAGCTGGGTTTCAATGCTGAAGCATCATCAT
SKS12-full length	F: GGGGACAAGTTTGTACAAAAAAGCAGGCTTTATGAAGGGAGGGGTTAAACTTTTG<br>R: GGGGACCACTTTGTACAAGAAAGCTGGGTTTTAAGCTCCGGCATAAGGGTT
SKU5-GPI overlap with SKS12	F: GTTAACCCTTATGCCGGAGCTTCGTCGGCATCGAAGAGC<br>R: GCTCTTCGATGCCGACGAAGCTCCGGCATAAGGGTTAAC

SKS12+SKU5-GPI	F: GGGGACAAGTTTGTACAAAAAAGCAGGCTTTATGAAGGGAGGGGTTAAACTTTTG<br>R: GGGGACCACTTTGTACAAGAAAGCTGGGTTTCAATGCTGAAGCATCATCAT
SKS17-full length	F: GGGGACAAGTTTGTACAAAAAAGCAGGCTTTATGAAAATGGCATCAAGAAAAACAACATC<br>R: GGGGACCACTTTGTACAAGAAAGCTGGGTTCTAAACATGTCGTCCAACAGCTTT
SKU5-GPI overlap with SKS17	F: CAAAGCTGTTGGACGACATGTTTCGTCGGCATCGAAGAGC<br>R: GCTCTTCGATGCCGACGAAACATGTCGTCCAACAGCTTTG
SKS17+SKU5-GPI	F: GGGGACAAGTTTGTACAAAAAAGCAGGCTTTATGAAAATGGCATCAAGAAAAACAACATC<br>R: GGGGACCACTTTGTACAAGAAAGCTGGGTTTCAATGCTGAAGCATCATCAT
irt1 mutant	IRT1-D1T1-BsF:<br>ATATATGGTCTCGATTGTCAACTGCGCCGGAAGAATGGTT<br>IRT1-D1T1-F0:<br>TGTCAACTGCGCCGGAAGAATGGTTTTAGAGCTAGAAATAGC<br>IRT1-DT2-R0:<br>AACTCTGGTTGGAGGAACGAAACCAATCTCTTAGTCGACTCTAC<br>IRT1-DT2BsR:<br>ATTATTGGTCTCGAAACTCTGGTTGGAGGAACGAAACC
SKU5-QRT-1	F: AATGTCAGAAACAAGTTAGACGAGGGA<br>R: GAGAAAGGGACTGGAATAATAGC
SKU5-QRT-2	F: CGCCTTCTTTGTCGTCGGTATGG<br>R: CCGACGATGAAACCTTCTGTGGC
SKS1-QRT-1	F: GTATCCAAATGCGGCGTAACTCG<br>R: GGGAAAGGGATAGGAATAATGTC
SKS1-QRT-2	F: CCAAGACGCCACAAGTGACTACTACAT<br>R: TGGTCTTCGGCTGGCTCATTGCT
SKS2-QRT-1	F: TCAGATGAGGCGTAACTCGTGGCA<br>R: TCAGGCTCAGTGAACGGAATAGG
SKS2-QRT-2	F: CTATCATCAACGCAACATACAAG<br>R: GGATAGACCTCCACCGTGCTTCG
SKS3-QRT	F: CGTTTTCTTGTCGGTTACCGGAGCC<br>R: CCAATTACCTGTTGACGAGTGCC
SKS12-QRT	F: GGGACATGGACACCAGAGAA<br>R: CGTACAGTTGCTGTCCCAAG
SKS17-QRT	F: GCACCAATGCCATTCAAAGC<br>R: ACCAAATCCAACGACCCAGA
RBOHA-QRT	F: CGTGTCATGTCCCATTTCGC<br>R: CTAACCCAGCTGCTCCACAA
RBOHB-QRT	F: AATTACTTCGGCTTCCGGGG<br>R: AGGGAAGTTGACAAACCTTGGA
RBOHC-QRT	F: CTCACCAGAGACTGGCACAA<br>R: TAGTCACCGCCTTGATGCTC
RBOHD-QRT	F: ACTCTCCGCTGATTCCAACG

	R: GACGTTATTCCGGCGAGCTA
RBOHE-QRT	F: AGACCTCGTCATGTGGTTCAAA<br>R: ATGGCCATAAGCATGACCCA
RBOHF-QRT	F: TCAGAGCCGACGAAACAACA<br>R: GCACCAATGCCAAGACCAAC
RBOHG-QRT	F: CATGCATCCAAAACAAGGCAGA<br>R: TTCCGCTTGCAGAAGCAATTT
RBOHI-QRT	F: TGCCCCTGAAGCTGGAAAAA<br>R: CTTCCAATGGTCTTGCGCTG
RBOHC-promoter	F: GGGGACAAGTTTGTACAAAAAAGCAGGCTTTGATCGAATTCGTCGTCGGTG<br>R: GGGGACCACTTTGTACAAGAAAGCTGGGTTTTTTTAACACACTCTACCTGAAAATTTTCTCG
sku5 (Flag_386B03) identification	LP: CTCGTGAATCCAATGCTCTTC<br>RP: CAAAACGCGAGCTCTGATTAC
sks1 (Flag_521F09) identification	LP: GAATGGAGACTCAACACAGGC<br>RP: TCGATTGAGAGATTCAATGGC
sks2 (Flag_607D01) identification	LP: TGTGGGACAGTCTTATTCTTTCCT<br>RP: ATAATCTCCTTTCACTTTATGGT
rbohc (SAIL_1275_E08) identification	LP: AGTGATGATAATGCAGGGTGG<br>RP: AACAAAACCATCACAAGTCCG
LB2	GCTTCCTATTATATCTTCCCAAATTACCAATACA
LB4	CGTGTGCCAGGTGCCCACGGAATAGT

Supplemental Table S3. Cloning Strategy.

Name	Promoter	Vector
p35S::SKU5-GFP	*p35S*	pK7WG2
p35S::SKU5- ΔGPI-GFP	*p35S*	PHB
pSKS1::SKS1-GFP	*pSKS1*	pBGWK
p35S::SKS1-GFP	*p35S*	pK7WG2
p35S::SKS1- ΔGPI-GFP	*p35S*	pGWB502
p35S::SKS3	*p35S*	pGWB502
p35S::SKS3+GPI	*p35S*	pGWB502
p35S::SKS12	*p35S*	pGWB502
p35S::SKS12+GPI	*p35S*	pGWB502
p35S::SKS17	*p35S*	pGWB502
p35S::SKS17+GPI	*p35S*	pGWB502
pRBOHC::GUS	*pRBOHC*	pGWB533
